# Phenylpyrrole fungicides act on triosephosphate isomerase to induce methylglyoxal stress and alter hybrid histidine kinase activity

**DOI:** 10.1038/s41598-019-41564-9

**Published:** 2019-03-25

**Authors:** T. Tristan Brandhorst, Iain R. L. Kean, Stephanie M. Lawry, Darin L. Wiesner, Bruce S. Klein

**Affiliations:** 1 Departments of Pediatrics, School of Medicine and Public Health, University of Wisconsin-Madison, Madison, WI 53792 USA; 2 Internal Medicine, School of Medicine and Public Health, University of Wisconsin-Madison, Madison, WI 53792 USA; 3 Medical Microbiology and Immunology, School of Medicine and Public Health, University of Wisconsin-Madison, Madison, WI 53792 USA

## Abstract

Fludioxonil, a natural product of pyrrolnitrin, is a potent fungicide used on crops worldwide. Drug action requires the presence of a group III hybrid histidine kinase (HHK) and the high osmolarity glycerol (HOG) pathway. We have reported that the drug does not act directly on HHK, but triggers the conversion of the kinase to a phosphatase, which dephosphorylates Ypd1 to constitutively activate HOG signaling. Still, the direct drug target remains unknown and mode of action ill defined. Here, we heterologously expressed a group III HHK, dimorphism-regulating kinase 1 (Drk1) in *Saccharomyces cerevisae* to delineate fludioxonil’s target and action. We show that the drug interferes with triosephosphate isomerase (TPI) causing release of methylglyoxal (MG). MG activates the group III HHK and thus the HOG pathway. Drug action involved Drk1 cysteine 392, as a C392S substitution increased drug resistance *in vivo*. Drug sensitivity was reversed by dimedone treatment, indicating Drk1 responds *in vivo* to an aldehydic stress. Fludioxonil treatment triggered elevated cytosolic methylglyoxal. Likewise, methylglyoxal treatment of Drk1-expressing yeast phenocopied treatment with fludioxonil. Fludioxonil directly inhibited TPI and also caused it to release methylglyoxal *in vitro*. Thus, TPI is a drug target of the phenylpyrrole class of fungicides, inducing elevated MG which alters HHK activity, likely converting the kinase to a phosphatase that acts on Ypd1 to trigger HOG pathway activation and fungal cell death.

## Introduction

Fungicides are used worldwide to combat fungal infection in agriculture and in human and animal disease. Fludioxonil, an agricultural fungicide of the phenylpyrrole class, is widely used on crops both pre- and post-harvest, originally finding applications as a preservative for seed storage^1,2^. Fludioxonil is a chemical derivative of the natural product pyrrolnitrin, initially isolated from *Pseudomonas*^2–4^. Despite extensive study and the discovery that the drug’s action requires the presence of group III hybrid histidine kinases (HHKs), its molecular target is unknown and mode of action incompletely understood.

Group III HHKs have been widely studied for their role in pathogenesis, morphogenesis, and fungicide sensitivity^5–7^. HHKs are sensor kinases that regulate environmental stress response pathways, such as the high osmolarity glycerol (HOG) pathway. Under non-stress conditions, the prototypical HHK from *Saccharomyces cerevisiae*, a group VI HHK called Sln1, is active and negatively regulates the HOG pathway^7–11^. Exposure to hyperosmotic or extra-cellular aldehydic stress, however, abrogates Sln1 activity, leading to phosphorylation of the Hog1 transcription factor and activation of the cell’s stress response elements^11,12^. When cells expressing group III HHKs are exposed to fludioxonil, Hog1 becomes constitutively activated leading to cell-cycle arrest, glycerol accumulation, cell swelling, and rupture^13–15^.

Group III HHKs like Nik1 of *Candida albicans* or Drk1 of *B*. *dermatitidis* are required for the fungicidal action of fludioxonil. When elements of the HOG pathway are deleted, fungal cells become resistant to fludioxonil^16,17^. Conversely, heterologous expression of a group III HHK in *S*. *cerevisiae*, which has no group lll kinase, renders it sensitive to fludioxonil^13,18,19^. Group III HHKs structurally diverge from the other classes of fungal HHKs due to the HAMP domain repeats at the N-terminus of the protein^8^. Importantly, when the HAMP repeats are deleted or modified so the HHK becomes a constitutive kinase, it no longer engenders fludioxonil sensitivity^20,21^.

We recently clarified the action of fludioxonil by showing that the drug induced the group III HHK Drk1 to act as a phosphatase and dephosphorylate its downstream phosphotransfer protein, Ypd1, *in vivo*^17^. Ypd1 dephosphorylation leads to Hog1 phosphorylation^10^. This finding revealed how group III HHKs act to inappropriately activate the HOG pathway in response to fludioxonil. We also showed that fludioxonil does not induce purified Drk1 protein to produce the same effect *in vitro*^17^, suggesting fludioxonil may not act directly on group III HHKs. This discrepancy between fludioxonil induced Drk1 behavior *in vivo* and *in vitro* led us to investigate whether group III sensor kinases alter their activity in response to a stress condition elicited by exposure to fludioxonil rather than by the direct action of the fungicide itself upon the HHK.

Herein, we heterologously expressed Drk1 in *S*. *cerevisiae* to (i) induce sensitivity to fludioxonil, (ii) investigate the drug target and mode of action, and (iii) decipher how Drk1 senses changes in intracellular homeostasis. Sensor kinases may rely on the reaction of signaling molecules with sentinel cysteine thiols rendered reactive by their chemical environment^22,23^. We hypothesize that Drk1 behaves as such a sensor, responding to drug-induced stress as one or more of its cysteine residues become modified. We furnish evidence to support this hypothesis through a mutational analysis of Drk1 cysteines, which enhances drug resistance. We tested intracellular stresses (e.g. nitrosation, oxidation, and glycation) that can modify cysteine thiols, and defined the intracellular target(s) that lead to their generation.

We report that the Drk1 HHK responds to aldehydic stress induced by elevated cytosolic methylglyoxal (MG) upon fludioxonil treatment. Elevated MG can result from blocked clearance by the glyoxalase system, build-up of a precursor dihydroxyacetone phosphate (DHAP, which is normally converted to glyceraldehyde 3-phosphate [G3P] by triosephosphate isomerase [TPI]), or allostearic interference with the active site of TPI thus promoting decomposition of the phospho-enediol intermediate to MG^24^. We show that fludioxonil treatment both inhibits TPI and causes it to convert triosephosphate into MG, likely by the latter mechanism. We offer a new model for the target and action of phenylpyrrole class drugs: upon modification of TPI function, group III HHK sense the engendered MG stress, which modifies the sensor at one or more cysteines converting it from a kinase to a phosphatase, inducing constitutive activation of HOG signaling and cell death.

## Results

### A cysteine mutation of Drk1 diminishes fludioxonil sensitivity

**Figure 1:**
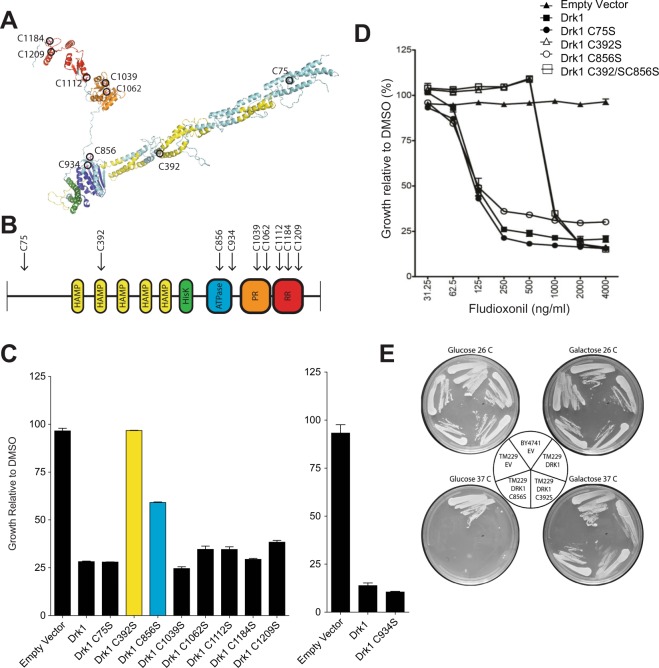
A Drk1 cysteine mutation decreases fludioxonil sensitivity in yeast. (**A**) Predicted 3-D structure of the group III HHK Drk1. Cysteine residues circled in black. Yellow: HAMP domains, green: Histidine kinase domain, blue: ATPase domain, orange: Pseudo-receiver domain, red: Response regulator/receiver domain. The “pseudo receiver” domain designation is based on the predicted structure. Though this domain lacks some of the specific amino acids residues to make it a true response regulator (or receiver) domain, it conforms to the response regulator tertiary structure: five β-sheets surrounded by five α-helices arranged in the exact order expected of a response regulator^87^. (**B**) Linear representation of Drk1 protein, highlighting the location cysteines and domains in which they are located. HisK, Histidine kinase domain; ATPase, ATPase domain; PR, Pseudo-receiver domain; RR, Response regulator, or receiver, domain. (**C** and **D**) *S*. *cerevisiae* BY4741 expressing empty vector (EV), wild type Drk1, or Drk1 with cysteine mutations was grown in a 96-well plate at 30 °C with 1 μg/ml of fludioxonil (**C**) or a range of fludioxonil concentrations (**D**) and 1% DMSO (solvent control). The OD^600^ of growth in fludioxonil is expressed as a percentage of the strain’s growth in DMSO alone. (Effect of mutation C934S evaluated in a separate experiment). EC^50^ and MIC values were: wild-type Drk1: EC^50^ = 125 ng/ml, MIC = 1000 ng/ml; C75S: EC^50^ = 125 ng/ml, MIC = 1000 ng/ml; C392S: EC^50^ = 825 ng/ml, MIC = 2000 ng/ml; C856S: EC^50^ = 125 ng/ml MIC = 2000 ng/ml. (**E**) *S*. *cerevisiae* BY4741, or TM229 containing temperature-sensitive Sln1 expressing EV, wild-type Drk1, Drk1 C392S, or C856S. Cells were grown on SC-Uracil medium with either glucose or galactose at the permissive temperature 26 °C or the non-permissive temperature of 37 °C.

We hypothesized that Drk1 cysteine thiols act as reactive “sentinels”^22,23^, responding to stress induced by fludioxonil treatment as one or more of these cysteines undergo modification. To test this premise, we mutated each of the Drk1 cysteines to serines, individually or in pairs, and determined the effect of these mutations upon fludioxonil sensitivity. Drk1 has nine cysteines, (Fig. 1A,B). We first screened the Drk1 cysteine mutants for resistance to a concentration of 1 µg/ml fludioxonil. Mutation of cysteines 392 and 856 increased fludioxonil resistance (Fig. 1C). We then tested these two single mutants, a C392S/C856S double mutant, and a control C75S mutant that did not exhibit increased fludioxonil resistance against a range of fludioxonil concentrations. The mutants gave different sensitivity profiles (Fig. 1D). The C856S mutant had the same EC^50^ as the wild-type Drk1 and control C75S mutant, although the growth profile may suggest delayed pathway activation at the highest concentrations of fludioxonil. The C392S mutant, on the other hand, had close to a one-log increase in EC^50^ compared to wild-type Drk1 and the control C75S mutant. At the highest concentrations of fludioxonil, however, this mutant showed the same (poor) growth as the wild-type Drk1, i.e., sensitive to the fungicide. A double C392S/C856S Drk1 mutant had the same resistance profile as the single C392S mutant (data not shown). These data suggest that fludioxonil sensitivity is mediated, at least in part, in a manner dependent upon Drk1 C392.

To ensure that a mutation of cysteine 392 did not alter the structure and function of Drk1 generally, we investigated its kinase activity. We transformed C392S Drk1 into a strain of *S*. *cerevisiae* harboring a temperature sensitive version of Sln1 (TM229). At a permissive (26 °C) temperature, Sln1 is functional, HOG1 signaling is inhibited, and yeast are viable. At a non-permissive (37 °C) temperature, Sln1 is inactive and yeast are inviable due to constitutive activation of HOG1 signaling^25^. A functional wild-type Drk1 kinase, conditionally expressed upon growth in galactose, rescues yeast lacking functional Sln1 (Fig. 1E lower right-hand plate). Likewise, conditional expression of either the C392S and C856S mutants of Drk1 rescued yeast grown at a non-permissive temperature of 37 °C. Thus, these Drk1 mutants retained sufficient kinase activity to inhibit constitutive HOG1 activation.

### A model for Drk1 sensing and conversion from kinase to phosphatase

**Figure 2:**
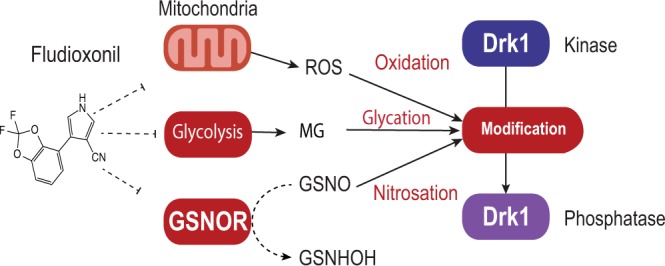
Potential modes of fludioxonil action through modification of HHK sensor kinase thiol(s). Drk1 is known to convert from a kinase to a phosphatase^17^, which may require modification (right), likely involving cysteines. Fludioxonil has been implicated in the collapse of electron gradients in mitochondria, which leads to the production of intracellular reactive oxygen species (ROS) that may modify Drk1 activity by oxidizing a cysteine thiol. Fludioxonil may inhibit nitrosoglutathione reductase, leading to the accumulation of nitrosoglutathione (GSNO), which can then nitrosate cysteine thiols. Finally, interference with glycolysis such that dihydroxyacetone phosphate (DHAP) accumulates may lead to the creation of methylglyoxal (MG), which can reversibly glycate cysteine thiols.

Sensitivity to modification of a chemically reactive thiol has been reported for many other sensor kinases^22,23^. Based on the effect that pyrrolnitrin has on mitochondrial electron transport chains, we postulated that Drk1 thiols might be sensitive to reactive oxygen species (ROS) within the cell. However, thiol reactive stress intermediates besides ROS could also act in this capacity. We thus tested several stress intermediates that could react with cysteine thiols, including ROS, nitrosating species and glycation (Fig. 2).

### Dismissal of elevated nitrosative stress, induction of glutathionylation and/or dimerization of thiols as modes of fludioxonil action

Despite fludioxonil’s behavior bearing some similarity to nitrosoglutathione reductase inhibitors^26^ (Supplementary Fig. S1), and possessing some inhibitory capacity of this target, we dismissed a nitrosative stress mechanism as the mode of fludioxonil action after generating substantial negative data (Supplementary Fig. S2). Based on an in-depth MS analysis of Drk1 isolated from fludioxonil-treated yeast, we also dismissed glutathionylation of sensitive cysteine residues and catalysis of disulfide formation as modes of fludioxonil action (Supplementary Fig. S3).

### Fludioxonil causes oxidative stress *in vivo*

**Figure 3:**
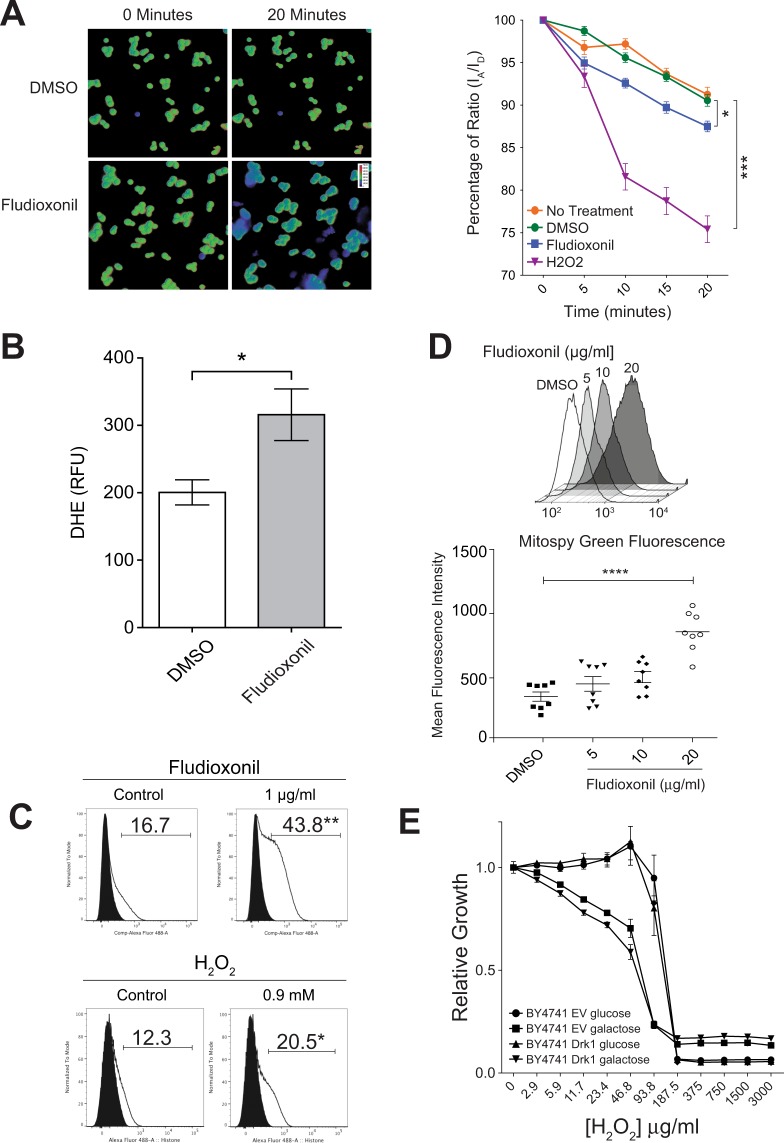
Fludioxonil induces oxidative stress *in vivo*. (**A**) YRF-1A yeast exposed to either 25 µg/ml fludioxonil, 1% DMSO (solvent control), or 1 mM H_2_O_2_ for 20 minutes. Left: FRET ratio image of FRET signal/CFP signal. Shift from green to blue indicates a decreased FRET ratio (oxidative stress). Right: The FRET ratio of 30 cells for each condition was measured every five min. for 20 min. Data are mean ± SEM. (**B**) DMSO was added to 1%, and fludioxonil in DMSO at 25 μg/ml and 1%, respectively. Dihydroethidium (DHE) was added to 20 μM. Incubation was for 1 hr at 30 °C with shaking. DHE fluorescence was recorded on a Filtermax F5 multi-mode microplate reader. (**C**) H2A.x phosphorylation of wild-type BY4741 yeast exposed to 1 μg/ml fludioxonil in 1% DMSO or 0.9 mM H_2_O_2_ for 60 min. The negative control for fludioxonil was 1% DMSO, the negative control for H_2_O_2_ was dH_2_O. Data are shown as percent of H2A.x positive cells, indicating oxidative stress. Gates were set using fluorophore minus one (FMO) gating. (**D**) Upper panel: Relative fluorescence of total cellular mitochondria in BY4741 cells exposed to fludioxonil in 1% DMSO. Representative single channel flow plots showing fluorescence intensity of cell population. Lower panel: Mean fluorescence intensity of total mitochondria per cell in *S*. *cerevisiae* BY4741 exposed to various concentrations of fludioxonil for 1 hour. Fluorescence was measured using flow cytometry. Error bars represent SEM from two experiments. (**E**) Broth dilution assay of *S*. *cerevisiae* BY4741 expressing Drk1 or empty vector (EV). Strains were exposed to H_2_O_2_ under induction (galactose) and repression (glucose) conditions, and relative survival was expressed as a ratio of growth compared to the no treatment control. Growth was measured by OD^600^. There was no significant difference in growth between Drk1 and EV cultures grown in galactose. For the above panels, *p < 0.05, **p < 0.01, ***p < 0.001, ****p < 0.0001. One-way ANOVA analysis with Bonferonni’s correction for multiple comparisons.

It has been conjectured that pyrrolnitrin causes oxidative stress in fungi due to its established effect upon the mitochondrial electron transport chain^27^. As fludioxonil is derived from pyrrolnitrin, we hypothesized that the drug might likewise induce oxidative stress. To test this, we employed the redox-responsive FRET reporter yeast, RedoxFluor^28,29^. This reporter responds to oxidative stress with decreased FRET signal. We used RedoxFluor yeast to measure redox environment in *S*. *cerevisiae* after exposure to DMSO, fludioxonil or H_2_O_2_ (positive control). We saw a decreased FRET signal in response to fludioxonil and H_2_O_2_, indicating oxidative stress in the yeast (Fig. 3A). An assay based on dihydroethidium oxidation (DHE) showed a similar increase in oxidation of DHE in yeast that were incubated in fludioxonil compared to DMSO vehicle (Fig. 3B).

We next used a complementary approach to monitor oxidative stress: histone H2A.x phosphorylation. Histone H2A.x is phosphorylated during DNA damage and serves as a marker of oxidative stress^30^. This method is used in the Environmental Protection Agency’s (EPA) ToxCast “toxicity forecaster” screen, which uses high-throughput bioassays to evaluate potentially toxic side effects of environmentally relevant small molecules^30,31^. Fludioxonil has been tested in the ToxCast system using HepG2 cells, a liver hepatocellular carcinoma cell line, and was found to increase H2A.x phosphorylation, indicating oxidative stress, at concentrations equal to or greater than 12.5 µM (~3.1 µg/ml) after 24 hours^30^. To see if fludioxonil induces this same response in fungi, we used wild-type *S*. *cerevisiae* to measure H2A.x phosphorylation following fludioxonil exposure. We observed evidence of oxidative stress induced by fludioxonil and the positive control, H_2_O_2_ (Fig. 3C). After fludioxonil exposure, 44% of the cells stained positive for H2A.x phosphorylation, compared to 17% with the vehicle control, DMSO.

To further assess whether fludioxonil induces oxidative stress in fungi, we examined a third oxidative stress marker: mitochondrial morphology. In the ToxCast system, compromised electron transport in mitochondria is quantified by a concomitant increase in mitochondrial mass. Mitochondrial biogenesis is an established cellular response to the type of electron-transport disruption associated with pyrrolnitrin^32^, which characteristically evolves ROS^33–35^. Using MitoTracker Green dye, we tested wild-type *S*. *cerevisiae* for mitochondrial changes during fludioxonil exposure. The dye accumulates in mitochondria, and, after exposure to fludioxonil, we detected a concentration-dependent, significant increase in total MitoTracker Green fluorescence in the yeast (Fig. 3D). Thus, fludioxonil induces mitochondrial mass accumulation consistent with oxidative stress in *S*. *cerevisiae* just as it does in human cell culture^36,37^.

### Oxidative stress does not substitute for fludioxonil treatment

We reasoned that if fludioxonil acted by inducing oxidative stress, we should expect to observe toxicity in Drk1-expressing yeast after the direct application of reagents that induce oxidative stress. This was not the case. Growth of yeast expressing Drk1 was not inhibited more than yeast carrying the empty vector upon exposure to H_2_O_2_ (Fig. 3E). Extensive MS analysis of Drk1 thiols found no evidence of thiol oxidation in fludioxonil-treated cells beyond that observed in control yeast treated with 1% DMSO alone (data not shown). Whereas oxidative modification events were observable via MS due to background levels of these modified thiols, no increase in these events was noted following fludioxonil exposure.

Though our work demonstrates that a substantial oxidative stress is induced by fludioxonil treatment, in a manner that is independent of the presence of the Drk1 kinase, fludioxonil toxicity does not hinge on the presence of oxidative stress molecules like hydrogen peroxide or superoxide. During our pursuit of evidence for oxidized thiols, we sought to “capture” intermediate forms of oxidized thiol that might be too labile to observe directly by treating yeast with dimedone^38^. Dimedone reacts with aldehydes and partially oxidized cysteine thiols like sulfenic acid. MS analysis demonstrated that there was no reaction of dimedone with oxidized thiols in Drk1. No increase in dimedone adducts was observable between fludioxonil treated yeast and controls treated with DMSO control (data not shown).

**Figure 4:**
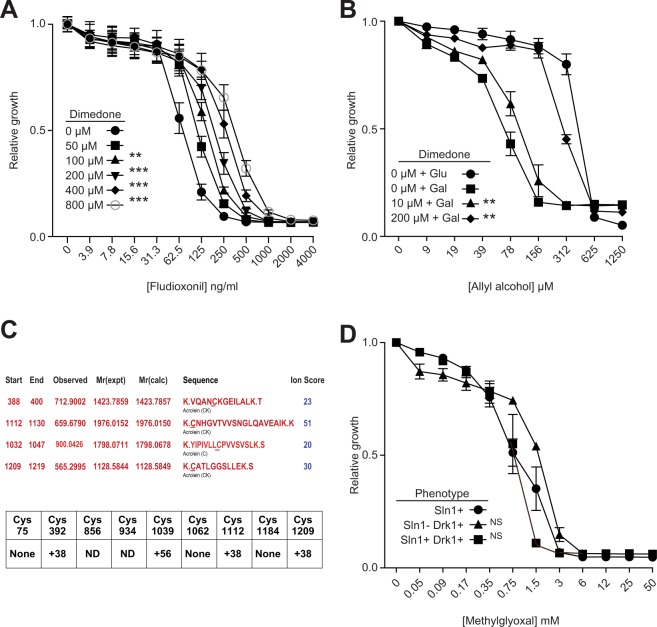
Dimedone protects against the effect of fludioxonil and allyl alcohol upon Drk1. (**A**) Growth assay measuring the effect of aldehyde binding molecule dimedone on fludioxonil killing of Drk1-expressing *S*. *cerevisiae* BY4741. Growth relative to DMSO control is shown. (**B**) Dimedone protection of allyl alcohol mediated killing of *S*. *cerevisiae* yeast-expressing Drk1. Growth relative to DMSO control is shown. (**C**) Tryptic fragments of Drk1 bearing masses derived from acrolein modification of cysteine thiols (+38 and +56)^42^. ND = fragment not detectable. (**D**) BY4741 (Sln1+) strain TM229 transformed with Drk1 and grown at a restrictive temperature (Sln1−Drk1+) or permissive temperature in galactose (Sln1+Drk1+) with the addition of methylglyoxal at concentrations indicated. Error bars are from 4 replicates. Significance is calculated between curves at maximum slope vs. control. **p < 0.01; ***p < 0.001; NS, not significant.

Interestingly, and unexpectedly, dimedone treatment was seen to attenuate the toxic effects of fludioxonil in Drk1-expressing yeast (Fig. 4A). This protective effect was significant and concentration dependent. This observed protective effect of dimedone was revealing, since this reagent is a standard treatment for the protection of cells against aldehydic stress (below). This dimedone effect could be replicated in a native, fludioxonil-sensitive strain of *Candida albicans* (K1). Thus, dimedone protected *C*. *albicans* against fludioxonil toxicity just as it did Drk1 expressing *S*. *cerevisiae* (Supplementary Fig. S4).

### Toxicity of fludioxonil on Drk1-expressing yeast can be duplicated by aldehydic stress

Treatment of yeast with allyl alcohol induces intracellular aldehydic stress in the form of acrolein^39^. Acrolein is an aldehydic toxin with well-established fungicidal properties. Yeast induced to express Drk1 exhibited more sensitivity to allyl alcohol compared to uninduced controls (Fig. 4B). This increased sensitivity could be reversed almost entirely through the addition of dimedone, in a concentration dependent manner. These results argue that the stress condition triggering the Drk1 kinase *in vivo* is likely an aldehydic stress.

Aldehydic stress can derive from various stimuli. The most common are aldehydes derived from the oxidative breakdown of cellular lipids^40,41^. With this in mind, we initially searched for adducts that form between lipid-derived aldehydes and cysteine thiols, using MS of PAGE-isolated Drk1 protein. The evidence for these known adducts forming in fludioxonil treated yeast, compared to controls, was entirely negative (data not shown).

Another, more elusive form of cellular aldehydic stress are aldehydes derived from a product of glycolysis, DHAP (dihydroxyacetone phosphate). DHAP can spontaneously convert into the damaging aldehyde MG, but while MG reacts rapidly with sensitive cysteine residues, this reaction may be quickly reversed. Searching for MG modifications directly was not fruitful, so we assessed reactivity of the Drk1 cysteine thiols through a surrogate stressor. We examined allyl alcohol treated yeast, employing nanoLC-MS/MS to seek adducts formed between the aldehyde acrolein and the protein thiols of Drk1 (mass of + 56 initially, converting to a more stable + 38 form over time^42^) (Fig. 4C). A tryptic digest of Drk1 provided sequence coverage of all but two cysteines (Supplementary Fig. S5). Four of the cysteine residues examined bore modifications with these masses in treated vs. untreated yeast (Supplementary Fig. S5). Modifications of two cysteines (C392 and C1112) were detectable with a relative abundance of ∼10–20% (Fig. 4C); two (C1039 and C1209) were modified at trace levels, and three (C75, C-1062 and C-1184) were unmodified. Drk1 thus becomes modified at specific, sensitive cysteine thiols during the response to this surrogate aldehydic stress. This is not inconsistent with MS experiments in which yeast treated with fludioxonil showed a depletion of reduced and DTT-reducible cysteines in the Drk1 HHK compared to DMSO-treated controls. Three cysteines (C75, C392 and C1112) showed significant (50–60%) reductions in the reduced thiol form, while one (C1209) showed no change (Supplementary Fig. S3). The specific mass/derivation of the DTT-resistant modifications that accumulated in response to fludioxonil was not resolvable.

Despite the fact that C392S mutations to Drk1 rendered *S*. *cerevisiae* significantly less sensitive to the effect of fludioxonil, this result was not observed in experiments in which the yeast were exposed to allyl alcohol (Supplementary Fig. S6) or MG (data not shown). These results suggest that, while cysteine 392 of the Drk1 HHK appears to be involved in the molecular response to fludioxonil, its reactivity is not solely sufficient to drive the mechanism(s) of response to aldehydic stress.

Wild-type *S*. *cerevisiae* bears Sln1, a group VI HHK, on its surface, which responds to osmotic stress, but also to extracellular aldehydic stress^43^. This makes direct application of exogenous MG problematic for distinguishing the response of Sln1 versus Drk1. To circumvent this issue, we engineered the TM229 strain, in which Sln1 is inactive at restrictive temperatures, to express Drk1 (Fig. 1E). Growth of this strain at the permissive temperature (26 °C) allowed us to probe and validate the native, Sln1-dependent sensitivity of the yeast to MG. By growing the yeast in galactose at 26 °C, Drk1 and Sln1 were expressed together, and by growing the yeast at the non-permissive temperature (37 °C) in galactose, we probed the capacity of Drk1 to respond to MG in the absence of native Sln1 activity. TM229-Drk1-expressing yeast grown at non-permissive temperature without galactose were non-viable (Fig. 1E), verifying that native Sln1 is inactive at elevated temperatures and glucose repression inhibits transcription of Drk1; thus, neither kinase suppresses activation of the HOG pathway (lethal, when constitutive). However, when the inactive Sln1 MG sensor was complemented by Drk1, yeast remained as sensitive to MG as wild-type yeast (Fig. 4D). This result establishes that MG treatment phenocopies fludioxonil treatment in *Saccharomyces* yeast and lends credence to the theory that Drk1 might serve as an intracellular MG sensor, just as Sln1 acts as a sensor of extracellular MG^43^. Sensitivity to MG was likewise recapitulated in a native, fludioxonil-sensitive strain of *C*. *albicans* (Supplementary Fig. S4).

### Fludioxonil inhibits TPI, the enzyme that controls DHAP

**Figure 5:**
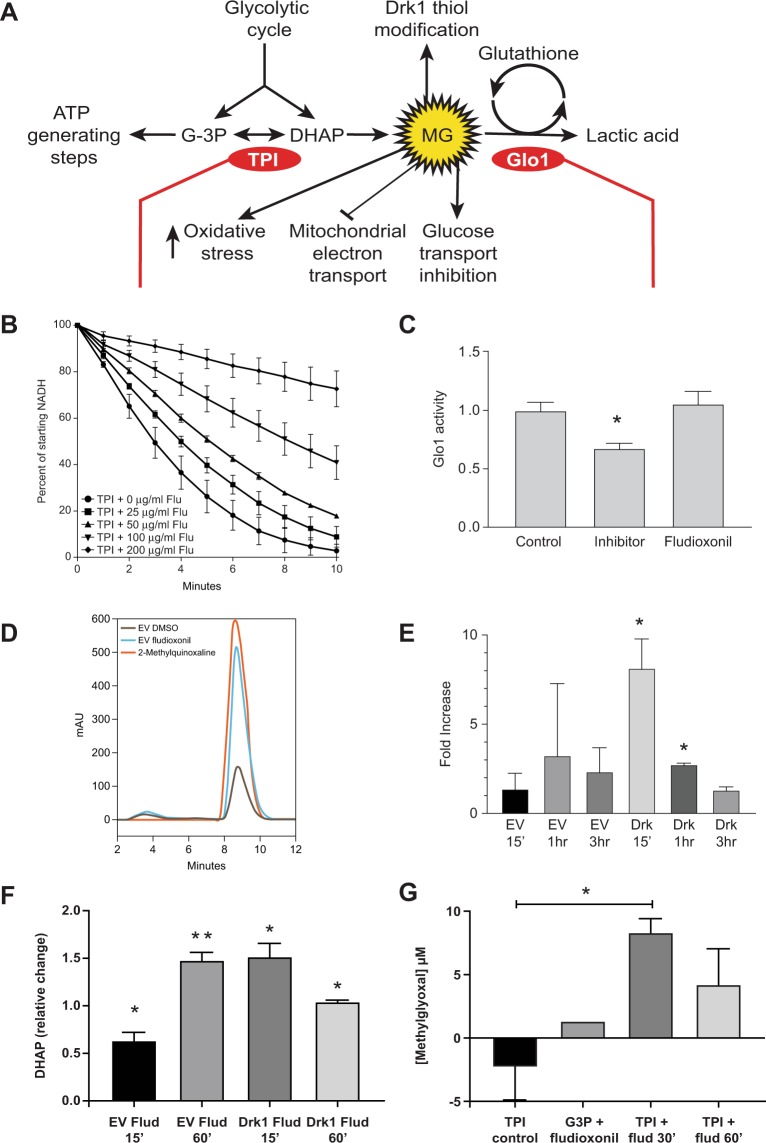
Fludioxonil inhibits the activity of triosephosphate isomerase (TPI) leading to elevation of methylglyoxal(MG). (**A**) MG concentration may be increased by inhibition of TPI, which acts upon a product of glycolysis, DHAP, or by inhibition of Glo1, which degrades MG with the assistance of a glutathione cofactor. (**B**) TPI was inhibited by incubation with fludioxonil for two hours at 37 °C. TPI activity was measured by NADH consumed by glycerophosphate dehydrogenase as it was processed from DHAP, which was generated by TPI from an excess of glyceraldehyde 3-phosphate. Loss of OD^340^ as NADH was consumed was monitored every 0.5 min. Data averaged from 4 replicates. (**C**) Glo1 was not inhibited by incubation with fludioxonil (10 mM in 1% DMSO) for two hours at 37 °C compared to a known glo1 inhibitor, S-hexylglutathione (10 mM in 1% DMSO). Components were incubated for 10 min. in 50 mM sodium phosphate buffer (pH 6.6) at 37 °C and OD^240^ monitored for comparison to starting value. (**D**,**E**) Cellular MG was measured by lysing cells at various time points after they were exposed to fludioxonil and measuring the 2-methylquinoxaline (MQ) adduct formed by the reaction of MG with 1,2-diaminobenzene. Quinoxalines were separated by RP HPLC and detected by absorbance at 220 nm. In panel D, pure MQ (red trace) is included as a positive control for time of elution from the HPLC column. (**F**) DHAP was quantified by fluorometric kit, which follows conversion of DHAP to G3P by linking the latter molecule to the activation of a fluorescent probe. (**G**) Release of MG by TPI was quantitated by running the reaction in the presence of 1,2-diaminobenzene and measuring formation of MQ adduct (by increase in OD^320^). *p < 0.05, **p < 0.01 vs. background or the respective control. One-way ANOVA analysis with Bonferonni’s or Tukey’s multiple comparison test.

For MG to accumulate in cells, one of two things must occur. Either one (or more) of the pathways that degrade MG (e.g. Glo1) must be inhibited^44^, or, alternatively, TPI must be prevented from converting the MG precursor, DHAP, to G3-P^45^ (Fig. 5A). We investigated the effect of fludioxonil on TPI activity and found dose-dependent inhibition with an IC_50_ of ~100 μg/ml (Fig. 5B). In contrast, fludioxonil had no effect on Glo1 activity (Fig. 5C). Thus, drug inhibition of TPI could potentially contribute to the elevation of MG levels seen in our yeast model of fludioxonil action.

### Yeast exposed to fludioxonil exhibit elevated levels of MG

Following the action of MG can be difficult - thiol modifications at sensitive “sentinel” thiols form rapidly, but appear to be transient, either reverting to reduced thiols just as quickly as MG concentrations drop^49,47^ or maturing into more complex adducts (of unknown mass). Glycation at cysteine thiols can also serve as an intermediate step in either transferring the modification to nearby amino groups^42^ or catalyzing the formation of disulfide bonds, thus a cysteine thiol may be critical to the function of an MG sensor kinase yet display no evidence of modification in MS analysis^48^. Fortunately, elevated cellular MG can be trapped as a 2-methylquinoxaline adduct via reaction with 2,4-diaminobenzene^49^. Increases in this adduct can then be measured by HPLC.

Wild-type yeast exposed to 0.1% DMSO vehicle alone showed a small elevation of MG (as 2-methylquinoxaline) over time, but when they were exposed to fludioxonil, cellular MG rose to much higher levels (Fig. 5D). The presence of Drk1 was not required for MG to accumulate. The peaks in Drk1-expressing yeast were actually smaller than in empty vector control-yeast at later time points (Fig. 5E), possibly because of the rapid rate at which fludioxonil renders yeast non-viable. Within 3 hours of fludioxonil exposure, viability in Drk1-expressing yeast is halved^26^. These results are consistent with a mechanism in which fludioxonil disrupts cellular homeostasis by inhibiting TPI, thus causing DHAP to accumulate.

### Fludioxonil alters the activity of TPI, causing the enzyme to convert triosephosphate directly into MG *in vitro*

To see if a model involving TPI inhibition was plausible, we looked at changes in the concentration of DHAP in response to fludioxonil vs. DMSO vehicle control. While small but significant changes in DHAP were noted (Fig. 5F), they seemed insufficient to explain MG levels that rose up to 10-fold (Fig. 5D,E). On this basis we concluded that TPI inhibition by itself was insufficient to explain the increases in cellular MG observed following treatment with fludioxonil.

Interference with the function of the loop 6 component of the TPI active site catalyzes the degradation of the triosphosphate intermediate into MG and free phosphate with increased exposure to solvent^24^. Since there is precedent for small hydrophobic inhibitors of TPI to act at the dimerization interface in a fashion that constrains this same loop^50^, we tested whether fludioxonil might induce TPI to release MG in this same way. We found that purified TPI treated with fludioxonil did convert a significant amount of triosephosphate substrate into MG, compared to background levels (Fig. 5G). This increase *in vitro* was more in keeping with the increases in MG we observed *in vivo*.

### Database docking study of fludioxonil at the dimerization interface of TPI

**Figure 6:**
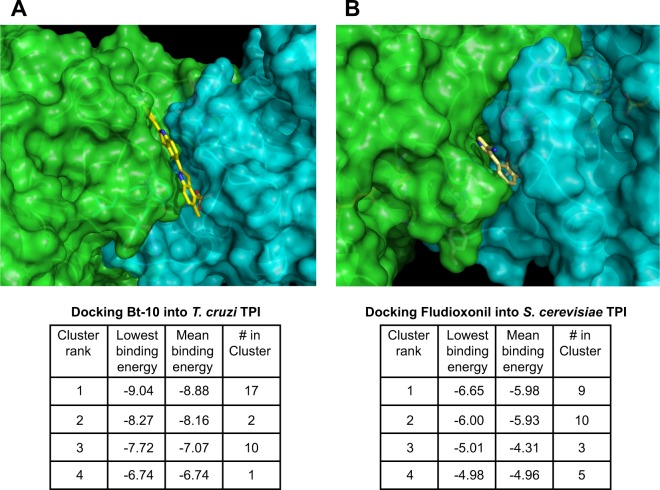
TPI inhibitors occupy the hydrophobic dimer interface region of TPI. Results of the top hits in the Autodock4 docking of (**A**) bt-10 in *T*. *cruzii* TPI and (**B**) fludioxonil in *S*. *cerevisiae* TPI (displayed in Pymol). Solvent accessible surface viewed along the interface between the dimerized TPI molecules (cyan) and (green) emphasizing the hydrophobic tunnel. The mean binding energy of −8.88 Kcal/mol for bt-10 in cluster 1 is lower than the mean binding energy of −5.98 Kcal/mol for fludioxonil because the ligand bt-10 contains a sulfate group H-bonded to a lysine residue in the protein, and has more atoms to contact the protein.

If fludioxonil acts upon TPI by binding within the hydrophobic tunnel region at the TPI dimer interface, as has been reported for TPI inhibitors like the benzothiazole derivative bt10^50^, docking studies for fludioxonil would be expected to show clustering within this same cleft. Our docking study of the bt10 molecule was consistent with the previous study^50^, with the molecule docking in the cleft and interacting with hydrophobic residues including Tyr103 in 1tcd (3D TPI structure from *Trypanosoma cruzii*) (Fig. 6A). Cluster analysis of 30 dockings gave the best cluster of 17 dockings having a top/average binding energy −9.04/−8.88 kcal /mol. For fludioxonil, 5 out of 30 were in the top cluster with a best/mean binding energy of −5.89/−5.81 Kcal/mol (In 1ypi, TPI from *S*. *cerevisiae*). Fludioxonil binds in the same cleft as bt10, interacting with homologous residues (Phe102) (Fig. 6B) but in a larger variety of orientations. The drug would be expected to yield a less favorable binding energy than bt10 because it is smaller, does not have the sulfate group driving specificity in bt10, and has a less favorable directionality. The binding energy of fludioxonil is, however, comparable to many smaller, benzothiazole TPI inhibitors^51^.

We postulate that these molecules act as steric effectors, inducing an inflexibility in loop 6 that permits the release of MG into the cytosol of our model yeast. This signal, in turn, converts the group III HHK of sensitive yeast into a phosphatase by modifying or activating one or more cysteine thiols, likely including C392. De-phosphorylation of Ypd1 leads to activation of Hog1 signaling, resulting in death of the yeast when this activation proceeds unabated.

## Discussion

Despite the worldwide application of fludioxonil to myriad agricultural products (>900), the mode of action of this fungicide is incompletely understood and its target is unknown. Due to the necessity of group III HHKs for sensitivity to fludioxonil, it was initially posited that these kinases fulfilled the role of direct target; this model went unchallenged for decades. Little data supported such a model and recent findings argue against direct drug action on HHKs^17^. Producers of the fungicide have since advanced a mode of action suggesting inhibition of transport-associated glucose phosphorylation^52^, a model that is not consonant with prior assumptions on direct action on HHKs. Here, we propose that the mechanism of fludioxonil is best explained by direct action upon TPI, which induces an unreported cytosolic stress – MG – that, in turn, triggers the activation of HHKs.

We show that fludioxonil modifies the action of TPI, inducing elevation in MG, an aldehydic stress. This drug target and mechanism is consistent with prior observations that fludioxonil treatment (i) impairs mitochondrial electron transport chains, (ii) reduces transport-associated phosphorylation of glucose^52^ and (iii) leads to accumulation of glycerol^53^. The first two effects can be a consequence of elevated MG^54–57^, whereas glycerol overproduction is promoted by an elevation in DHAP^45^. Thus, our data support a novel drug target and model in which the group III HHK Drk1 functions as a sensor of aldehydic stress intermediates.

The sensitivity of sensor kinases often hinges on modification of highly reactive “sentinel” cysteine thiols^58^. To test our hypothesis that fludioxonil affects Drk1 function indirectly via interaction of a stress-derived chemical signal with one or more cysteine residues, we tested fludioxonil sensitivity in *S*. *cerevisiae* bearing mutations in heterologously expressed Drk1. Mutants were created in which each of the 9 cysteine residues of Drk1 was replaced with a serine residue. One mutant, C392S, resulted in a log-fold increase in fludioxonil resistance. This partial effect suggests that C392 plays a role in drug sensitivity, but is insufficient by itself to mediate sensitivity, as this mutant remained sensitive to high concentrations of the fungicide and was as sensitive as the yeast with unmutated Drk1 to allyl alcohol-derived acrolein. This is not unexpected, as there has long been evidence that the mechanism of fludioxonil toxicity is multi-factorial and unlikely to be simplified to a single signaling event. For example, in the fungus *Botrytis cinerea*, the Hog1 pathway is surprisingly uninvolved in the organism’s sensitivity to fludioxonil^59^ and other investigators have reported that stress response pathways are critical to fludioxonil resistance, potentially complementing osmoregulation mechanisms^60^.

Drk1 could possess two or more sensitive cysteine residues that respond to different concentrations of fludioxonil, possibly dependent upon the redox environment created by the amino acids surrounding them. By this model, C392 might respond to stress signal molecules engendered by a low concentration of fludioxonil, but would be superseded by the response of one or more of the other cysteine residues at higher concentrations. For example, the Yap1 transcription factor in *S*. *cerevisiae* responds to MG via cysteine modification^47^. Yap1 can translocate to the nucleus so long as it possesses even one of its 6 cysteines. It is not until all 6 cysteines are mutated that Yap1 loses its capacity for nuclear localization in response to MG.

While mutations such as C392S could potentially lead to mis-folding of enzymes that compromise their function, we verified that wild-type Drk1 and the C392S mutant were capable of rescuing a lethal, temperature-sensitive mutation of Sln1 that renders yeast non-viable at elevated temperature^25^. Thus, kinase activity of Drk1 is active in *Saccharomyces* indicating that our cysteine substitutions did not abrogate that function.

The fact that C392 resides in a HAMP domain reinforces the idea that C392 is involved in sensing or signaling. The means by which group III HHKs sense and respond to fludioxonil involves HAMP domains. Since group III HHKs are required for fludioxonil sensitivity, others have hypothesized that HAMP domain repeats unique to group III HHKs are involved in sensing fludioxonil^8^. Of fludioxonil resistant mutants isolated in the field or laboratory, nearly all contain mutations in HAMP domains^2,61–63^. The HAMP domain region is also involved in regulating kinase activity of other group III HHKs^19,21^. Thus, it is not surprising that a cysteine in the HAMP domain region is involved in sensitivity to fludioxonil.

After fludioxonil exposure *in vivo*, the group III HHK Drk1 converts from a kinase, which phosphorylates its downstream target Ypd1, into a phosphatase^17^. *In vitro*, however, fludioxonil does not induce this change in Drk1 function, suggesting that the drug may not directly act on Drk1. These data, together with the observation that lead compounds identified in our drug screen share structural similarities with certain GSNOR inhibitors, led us to initially ask if fludioxonil acts as a GSNOR inhibitor, increasing intracellular GSNO. Our data showed that while fludioxonil weakly inhibited GSNOR this inhibition does not appear to be a significant element of fludioxonil’s mode of action. Additionally, neither SNO levels nor protein nitrosation increased intracellularly during fludioxonil exposure. Thus, nitrosation of Drk1 thiols is unlikely to be relevant in fludioxonil’s action.

Cysteine modifications are key elements in detection and response to oxidative stress^64,65^. Based on pyrrolnitrin’s propensity to block mitochondrial respiration^32,66,67^, a capacity to induce oxidative stress is plausible. We used multiple techniques to establish that fludioxonil induces oxidative stress *in vivo*. We also sought evidence for modifications catalyzed by this stress. For instance, S-glutathionylation of cellular proteins is higher under conditions of oxidative stress^68^. We thus investigated whether glutathionylation of sensitive cysteine residues proceeds from fludioxonil treatment; however, evidence of glutathionylation of Drk1 in response to fludioxonil *in vivo* was lacking, either at cysteine residue 392 or any other residue.

Exposure of fludioxonil-sensitive yeast to different oxidative stress inducing molecules, such as H_2_O_2,_ failed to phenocopy the effects of fludioxonil. Examination of Drk1 protein from fludioxonil-treated and untreated yeast using nanoLC-MS/MS also failed to detect oxidized forms of its cysteine thiols. Even though fludioxonil triggers oxidative stress, this stress is not sufficient, by itself, to mediate the toxicity of the drug.

During our search for oxidized cysteine residues, we treated yeast with dimedone to react with, and mass-label, any labile sulfenic acid groups that formed. No such groups were detected by MS, but we did find that dimedone protected yeast against fludioxonil toxicity. Dimedone is a known protectant against aldehydic reactants, and this suggested that the action of fludioxonil on yeast could involve aldehydic stress. We found that allyl alcohol, which induces intracellular aldehydic stress, phenocopied the toxicity of fludioxonil, and this effect was blocked by dimedone just as it countered the effect of fludioxonil. This data establishes that the toxic effect fludioxonil has upon sensitive fungi may be reversed by a reagent that blocks aldehydes.

In wild-type yeast, Sln1 on the surface is sensitive to extracellular aldehydes. Activation of Sln1 with exogenous MG over-stimulates the HOG pathway, just as fludioxonil kills fungi through activation of the group lll HHKs and constitutive HOG signaling^43^. We found that a temperature sensitive Sln1 mutant remained highly sensitive to MG at Sln1 non-permissive temperature, when rescued by Drk1. This established that Drk1 also responds to this aldehydic stress.

Since oxidative stress accompanies fludioxonil exposure, and oxidative intermediates can convert cellular lipids into aldehydic species, we sought evidence of these in the form of known adducts derived from their reaction with cysteine thiols. NanoLC-MS/MS analysis of Drk1 failed to find adducts formed from a reaction with acrolein or any other lipid-derived aldehydes, making it unlikely that these were the source of aldehydic stress. We theorized that fludioxonil might instead evolve aldehydic stress in the form of MG by disrupting one of the enzymes responsible for its normal metabolism. Under normal circumstances, TPI isomerizes the MG precursor, DHAP, into G3-P, minimizing MG creation, while Glo1 is the enzyme responsible for the breakdown of MG into (significantly less harmful) lactic acid. We thus investigated the capacity of fludioxonil to inhibit TPI and Glo1, discovering that it did inhibit the former, but not the latter.

TPI inhibition engenders a known toxic effect in actively metabolizing cells. This toxicity is sufficiently strong that TPI inhibitors exercise a chemotherapeutic effect and new TPI inhibitors are sought to treat cancer and parasitic infections^69,70^. In a small molecule screen for TPI inhibitors^71^, a frequently seen structural motif was the linkage of a benzyl group to a substituted pyrrole by a single carbon-carbon bond such that the aromatic structures may achieve a co-planar configuration. This configuration allows the molecules to fit into the narrow, hydrophobic tunnel at TPI’s dimerization interface. Fludioxonil’s structure is comprised of these same features. Of potential interest is the fact that beta-carbolines that inhibit TPI also display a fungicidal effect^72^.

Were fludioxonil to inhibit TPI, exposed cells would accumulate the MG precursor DHAP, which is normally maintained at low concentrations by the action of TPI. Since DHAP converts to MG spontaneously, TPI inhibition would be predicted to drive up the concentration of MG in affected cells. We found that MG levels increased in *S*. *cerevisiae* in response to fludioxonil. In cells expressing Drk1, this increase waned at approximately the rate that yeast lost viability^26^. The increase in DHAP levels, however, was not commensurate with the increase in MG. TPI inhibition alone therefore did not adequately explain the acute spike in MG observed in our yeast model of fludioxonil activity.

Mutations in loop 6 of the TPI active site greatly increase the rate at which the triosephosphate substrate is released prematurely, degrading into MG and free phosphate^24^. Certain small, hydrophobic inhibitors can also bind to the dimerization interface of TPI in a way that modifies the mobility of loop 6^50^. We reasoned that fludioxonil, a small, hydrophobic molecule, might work the same way, not only inhibiting TPI, but accelerating the generation of MG by a previously unreported mechanism. Indeed, our *in vitro* MG assay showed that approximately 2% of the triose processed by TPI was released from the enzyme as MG. This amount is greatly inflated compared to the normal rate, which is estimated to be 0.3% in yeast systems^73^. Our docking analysis showed that binding of fludioxonil to the hydrophobic tunnel at the dimeric interface of TPI is thermodynamically favorable, and comparable to the binding by known inhibitors of *T*. *cruzii* TPI to the same region in that enzyme^51^.

MG inhibits the electron transport pathway of mitochondria at complex 1^54^, causing oxidative stress to build-up^74^. It also suppresses the transport-associated phosphorylation of glucose^57^, just as the phenylpyrroles do^52^, and excess DHAP leads to the substrate-driven overproduction of glycerol^45^. These effects are also hallmarks of fludioxonil toxicity. Our results suggest they are side effects of the primary mechanism by which fludioxonil exerts its fungicidal effect e.g. a spike in cytosolic MG.

In summary, we propose that fludioxonil exerts its drug effect as follows. Fludioxonil modifies the activity of TPI, causing release of MG into the cytosol. MG modifies one or more cysteine thiols of group lll HHKs, converting it to a phosphatase, which dephosphorylates Ypd1, setting off a cascade that activates the HOG1 pathway constitutively, resulting in cell death.

Though our primary focus in this work has been to ascertain the mechanism by which fludioxonil causes the Drk1 HHK to turn into a phosphatase, it is also important to note that the fungicide fludioxonil has been deemed safe for application to food post-harvest by the EPA, and at fairly high concentrations, because it was believed to act directly upon group lll HHKs which human beings lack. If its actual target is TPI, fludioxonil could exert an insidious and difficult to detect toxic effect upon human cells. In fact, elevated MG is co-morbid with countless human disease states and its effect upon cells is difficult to distinguish from the natural effects of aging. As several other research groups have concluded^75,76^, and as summarized in our own review^77^, it is critical that the effects of fludioxonil upon human beings be re-evaluated at this time.

## Methods

### Strains, vectors, and growth conditions

The strains and vectors used in this study are listed in Tables S1 and S2, respectively. *S*. *cerevisiae* was grown in either yeast peptone dextrose (YPD) or synthetic complete (SC) media^78^ lacking uracil with either 2% glucose, 2% galactose/1% raffinose (induction media) or 2% raffinose (overnight growth) as the carbon source. *S*. *cerevisiae* cells were grown at 30 °C either stationary (96 well plate assays) or shaking at 200 rpm in Erlenmeyer flasks. To make the YRF-1A RedoxFluor strain, we first digested the pYRF-1A plasmid with MfeI and transformed 0.5 µg of linear plasmid into *S*. *cerevisiae* BY4741 and plated on SC-ura + glucose. *Candida albicans* strain K1 was grown on YPD medium.

### *E*. *coli* and *S*. *cerevisiae* transformation

*E*. *coli* was transformed by electroporation^79^. *S*. *cerevisiae* was transformed using the LiAc/SS carrier DNA/PEG method with the addition of 10 mM DTT^80^.

### Mutagenesis of Drk1 cysteine residues

Eight of the nine Drk1 cysteines were mutated using the QuickChange Multi Site-Directed mutagenesis kit (Agilent) with primers SML166-168, 170–174 (Table S3) and pYES-DEST52 Drk1 as template. Following mutagenesis, all sequences were confirmed with BigDye Terminator v3.1 mix (Applied Biosystems). Samples were cycled (96 °C for 2′, then 35 cycles of 96 °C for 10″, 52 °C for 15″, 60 °C for 3′, followed by 1′ at 72 °C) and cleaned up using CleanSeq magnetic bead sequencing reaction clean up kit (Agencourt, Bioscience). The samples were sequenced at the Univeristy of Wisconsin Biotechnology Center (UWBC).

For C934, when we employed the above method, we only recovered C934S mutants that had extra, unwanted mutations (insertions, deletions, or rearrangements). Considering the possibility of a toxic mutation, we mutagenized initially with a non-functional Drk1 (Drk1 D1140N^17^). The Drk1 C934S mutant was created by performing SDM as above with pYES-DEST52 Drk1 and SML169. The D1140N mutation was reverted by PCR with primers IRK099 and IRLK100 using Phusion polymerase (NEB) and sequence-confirmed mutants pYES-DEST52 Drk1 C934S D1140N. Linear DNA was phosphorylated with PNK enzyme (NEB) and self-ligated with T4 DNA ligase (NEB) in the same reaction. Ligation products were transformed into *E*. *coli* DH10B by electroporation. Mutants were confirmed by Sanger sequencing. The C392S/C856S double mutant was made using pYES-DEST52 Drk1 C392S as the template and primer SML168 to mutate the C856 to serine.

### GSNO synthesis

To synthesize GSNO, 620 µl 12 M HCl (Fisher) was added to 5.9 ml of water in a fume hood in the dark on ice with stirring. To this solution, 1.54 g GSH was added and allowed to dissolve, and then 1 ml of 0.5 M NaNO_2_ was slowly added and stirred for five minutes. The reaction was neutralized with 710 µl of freshly made 10 M NaOH and brought to 10 ml with 18.2 MΩ water. The concentration of GSNO was determined by measuring the absorbance at 335 nm and calculated using a millimolar extinction coefficient of 0.92. GSNO stocks were stored at −80 °C. All experiments involving GSNO, including production, were performed in the dark.

### NIH Small Molecule Screen

We undertook a screen with the National Institutes of Health (NIH) designed to discover small molecules that worked in a similar way to fludioxonil, i.e. compounds that killed fungi in a Drk1- and Hog1-dependent manner. We noticed that some of the hits that emerged from this screen were structurally similar to S-nitrosoglutathione reductase (GSNOR) inhibitors (Fig. S1). This led us to initially question whether fludioxonil might also act as a GSNOR inhibitor.

### GSNOR activity assay

To determine the effects of fludioxonil on GSNOR, we added 50 µl of 2 mM NADH (Sigma) (0.2 mM final concentration) to 425 µl GSNOR reaction buffer (20 mM Tris pH 7.5, 0.5% Deoxycholic Acid (Sigma), 1.6 µg/ml purified GSNOR [Proteintech] or 10% murine liver lysate, 1% DMSO ± the indicated concentration of fludioxonil) and incubated for seven minutes. The liver lysate was prepared by homogenizing fresh murine liver from C57BL/6 mice using a rotor-stator homogenizer, pelleting insoluble matter, and passing supernate through a 0.22 µm filter. Deoxycholic acid was used to solubilize fludioxonil. The initial absorbance at 340 nm (0 minute time point) was measured using a Cary 60 Spectrophotometer (Agilent), after which 25 µl GSNO was added to a final concentration of 0.4 mM and mixed by pipetting to start the reaction. Reactions were performed in triplicate. The absorbance at 340 nm was measured every 60 s for 3 minutes. A decrease in A340 (caused by a decrease in NADH) indicated GSNOR activity. We calculated the change in A340 from 0 minutes to 3 minutes. The relative activity was determined by normalizing the ΔA340 for the fludioxonil samples to the ΔA340 of the control.

### *S*. *cerevisiae* BY4741 *ΔSfa1* (GSNOR) assay

A strain of *S*. *cerevisiae* containing a disruption of the *sfa1* gene was transformed with pYES-DEST52, or pYES-DEST52 Drk1. Cultures were grown overnight at 30 °C with shaking in 10 ml SC-Ura + 2% raffinose. Fresh 10 ml cultures were inoculated to an OD600 of 0.1 in SC-Ura with 2% glucose, or 2% galactose and 1% raffinose. Fludioxonil was added to a final concentration of 25 µg/ml in 1% DMSO. Control cultures contained 1% DMSO. The cultures were grown at 30 °C overnight with shaking, and OD600 was measured after 24 h.

### Microtiter dilution assays

Overnight cultures of yeast grown in SC-ura + raffinose were passaged to an OD600 of 0.025 in SC-ura + glucose or SC-ura + galactose. Standard MIC dilution broth testing procedures were used with the above media. The final volume in each well was 200 µl. Plates were incubated at 30 °C for 48 hours. Yeast were suspended by shaking prior to OD600 measurement. The growth was calculated as a percentage of the growth relative to DMSO alone.

### Nitric oxide analysis

A wild-type yeast culture grown overnight in YPD was diluted to OD600 of 0.4 and grown for another 2 hours at 30 °C. 5 mM GSNO and either 1% DMSO, 25 µg/ml fludioxonil, or 25 µg/ml N6022 was added to 25 ml of culture and the culture was grown another 2hrs at 30 °C, while shaking. Cells were centrifuged and the cell pellets were stored at −80 °C. To lyse cells, pellets were resuspended in 500 µl Yeast Lysis Buffer (50 mM Tris-HCl, pH 8.0, 1 mM DTPA, 5 mM NEM, 0.1% Triton X-100) and transferred to a 2 ml tube containing 0.5 g of 0.5 mm acid-washed glass beads. Tubes were vortexed for 30 seconds followed by 30 seconds on ice for a total of 10 minutes (5 minutes of total vortex time). Crude lysates were centrifuged for 10 minutes at 10,000 rpm and 4 °C to pellet unbroken cells and insoluble material.

The clarified lysates were transferred to a new tube and stored at −80 °C. Protein concentration was determined with a BCA Protein Assay Kit (Pierce). S-nitrosothiol (SNO) levels were measured using the tri-iodide-dependent ozone-based chemiluminescence method, as previously described^81^. Briefly, 0.1 volume of 30 mM sulfanilamide in 2 N HCl was added to each sample, shortly before analysis, to remove nitrite. 10–20 µl of sulfanilamide-treated lysate was then injected into a Sievers 280i Nitric Oxide Analyzer vessel containing 5 ml of the I_3_^−^ reducing agent (14 mM I_2_, 33.4 mM KI, 77.7% acetic acid) made fresh that day. The amount of SNO was measured as area under the readout curve (mV over time) and normalized to the amount of protein in the lysate.

### Drk1 predicted structure modeling and domain identification

The 3D structure of Drk1 was predicted using the RaptorX Structure Alignment server (http://raptorx.uchicago.edu/)^82^. The images were constructed in MacPyMOL (Schrödinger, LLC) using the output from RaptorX. Drk1 domain designations were from the results of the NCBI Conserved Domain Database^83^.

### FRET assay

The YRF-1A RedoxFluor strain was grown overnight in SC-ura + glucose, backpassed into the same medium, and grown to around OD600 of ~0.9. 0.5 ml of yeast was then placed onto Concanavalin A (ConA)-coated 35 mm glass bottom dishes (MatTEK, #P35G-1.5-14-C). Yeast were allowed to adhere for 2–3 minutes and unadhered yeast were removed. The dishes were carefully washed twice with media and fresh media added to each dish. A 0 minute reading was taken on an Olympus IX83 microscope with a Semrock CFP/YFP/HcRed-3 × 3M-B-000 filter, Hamamatsu ORCA Flash 4.0 camera, and X-Cite 120PC light source, all controlled by the MetaMorph Software platform. The CFP signal was read first (427 nm excitation/472 nm emission) followed by the FRET signal (427 nm excitation/542 nm emission). After taking the 0 minute image, water (no treatment), 1 mM H_2_O_2_, 1% DMSO, or 25 µg/ml fludioxonil (in 1% DMSO) was immediately added to the dish. Images were taken every five minutes for 20 minutes total. To obtain the composite image, both sets of images were background subtracted and then the FRET image was divided by the CFP image^84^ (Fiji, ImageJ). For quantification, a 5 × 5 square was drawn around 30 cells in each image and the intensity in that square was measured^84^ (Fiji, ImageJ). The FRET signal (I_A_: Intensity of the acceptor) was divided by the CFP signal (I_D_: Intensity of the donor). I_A_/I_D_ ratios were normalized to the starting 0 minute value. ConA-coated dishes were prepared by adding 0.5 ml of 1 mg/ml ConA (in sterile dH_2_O) to the center of the dish and incubating the dish for 5 minutes at room temperature. The unadhered ConA was removed, and the dishes were washed twice with sterile dH_2_O and allowed to dry. Coated dishes were stored at 4 °C for a maximum of two weeks before use.

### H2A.x phosphorylation assay

Wild-type BY4741 *S*. *cerevisiae* yeast were grown overnight with YPD and backpassed into fresh YPD to an OD600 of 0.3–0.5. Cultures were grown another 4–5 hours and were split into treatment groups. dH_2_O, 1% DMSO, or the indicated concentrations of either H_2_O_2_ (in dH_2_O) or fludioxonil (in 1% DMSO) were added and cells were grown for 1 hour. Cells were collected by centrifugation and suspended in 1 ml of KS buffer (0.1 M Potassium phosphate buffer pH 7.4, 1 M sorbitol) with 3% formaldehyde and incubated for 15 minutes at 30 °C, with shaking. 3% ethanolamine was added and incubated with shaking for 15 min at 30 °C. Cells were then transferred to a deep well microtiter plate where they were washed twice with KS buffer. Cells were collected by centrifugation and suspended in 1 ml KS buffer after which 2.5U/ml Zymolase (Zymo Research) (in KS buffer) was added to cells and incubated for at least one hour at 37 °C to digest the cell wall, followed by washing with KS buffer. Cell permeabilization was performed by suspension and incubation in 500 µl of ice-cold methanol for 5 min at −20 °C. Cells were then incubated in 500 µl of ice-cold acetone and incubated for 30 seconds at −20 °C. After permeablization, cells were washed twice with KS buffer. Blocking was performed for one hour at 4 °C in PBS containing 10% normal Donkey serum. Cells were incubated with 1:1000 anti-γH2A.x antibody (EMB-Millipore Rabbit anti-γH2A.x [Ser129], #07-745-I) in PBS with 10% normal donkey serum at 4 °C overnight. Cells were washed twice with PBS and incubated with 1:500 anti-rabbit secondary antibody (donkey-anti-rabbit AF488, Jackson Labs) in PBS with 10% normal donkey serum and washed twice in PBS with 1% BSA. Cells were strained through 40 μm mesh immediately prior to cytometric analysis. The cells were analyzed for H2A.x phosphorylation with a BD LSR II flow cytometer and the data was processed with FlowJo.

### Enzymatic “In gel” digestion for mass spectrometry and *NanoLC-MS/MS*

All work was done by the Mass Spectrometry facility (UWBC) in accordance with their standard protocols for these techniques. https://www.biotech.wisc.edu/services/massspec/protocols/ingelprotocol.

Peptides were analyzed by nanoLC-MS/MS using the Agilent 1100 nanoflow system connected to a hybrid linear orbitrap mass spectrometer (LTQ-Orbitrap Elite™, Thermo Fisher Scientific) equipped with an EASY-Spray™ electrospray source. Chromatography of peptides prior to mass spectral analysis was accomplished using capillary emitter column (PepMap® C18, 3 µM, 100 Å, 150 × 0.075 mm, Thermo Fisher Scientific) onto which 3 µl of extracted peptides was automatically loaded. NanoHPLC system delivered solvents A: 0.1% (v/v) formic acid, and B: 99.9% (v/v) acetonitrile, 0.1% (v/v) formic acid at 0.50 µL/min to load the peptides (over a 30 minute period) and 0.3 µl/min to elute peptides directly into the nano-electrospray with gradual gradient from 3% (v/v) B to 20% (v/v) B over 18 minutes and concluded with 5 minute fast gradient from 20% (v/v) B to 50% (v/v) B at which time a 4 minute flash-out from 50–95% (v/v) B took place.

### MitoTracker Green staining of mitochondria

Cultures of wild-type BY4741 *S*. *cerevisiae* grown in YPD overnight were passaged to fresh YPD to OD600 0.3–0.5 and grown another 4–5 hours. The culture was divided and dH_2_O (no treatment), 1% DMSO or the indicated concentrations of fludioxonil (in 1% DMSO) was added to each aliquot. Cells were grown for 30 minutes, after which 100 nM MitoTracker Green (ThermoFisher) was added. Cultures were grown for a further 30 minutes. Cells were collected by centrifugation and suspended in KS buffer with 3% formaldehyde. Cells were washed twice with PBS, collected by centrifugation and suspended in PBS with 1% BSA. Cells were strained through 40 μm mesh immediately prior to cytometric analysis. MitoTracker Green fluorescence was measured with a BD LSRFortessa flow cytometer and the data was analyzed with FlowJo.

### Triosephosphate Isomerase inhibition assay

Triosephosphate isomerase activity was measured by the technique of Esnouf^85^, linking the isomerization reaction to an NAD dependent enzymic reaction. Briefly, we treated 5 μg of commercial Triosephosphate isomerase (from *S*. *cerevisiae*, Sigma) in 20 μl triethanolamine-HCl buffer (25 mM, pH 7.9) with 0, 25, 50, 100, and 200 μg/ml fludioxonil and 1% DMSO or with DMSO alone for 30 minutes prior to beginning assay. To a cuvette with a 1-cm light path, we added and mixed 1.4 ml of triethanolamine-HCl buffer, 50 μl of glyceraldehyde phosphate (10 mM), 25 μl of NADH (disodium salt, 7 mM), and 5 μl of α-glycerophosphate dehydrogenase (10 mg/ml). The reaction was started by adding the triosephosphate isomerase/inhibitor combination. The decrease in absorbance of the solution at 340 nm was monitored at 25 °C for 10 minutes on a Genesys 20 spectrophotometer (Thermo Scientific). Inhibition is inversely correlated with the ratio of (final OD340-background)/(starting OD340-background).

### Glo1 inhibition assay

Glo1 inhibition by Fludioxonil and the positive control inhibitor S-hexylglutathione was measured by a glo1 assay kit (MAK114, Sigma). Samples were exposed to either fludioxonil in 1% DMSO (10 mM) or S-hexylglutathione in 1% DMSO (10 mM) for 2 hours before the activity of glo1 was measured by production of S-lactoylglutathione from methylglyoxal and glutathione (followed by an increase in absorbance at 240 nm). Samples were read on a Filtermax F5 multi-mode microplate reader (Molecular devices).

### HPLC analysis of methylglyoxal in yeast by measurement of diaminobenzene adduct

BY4741 *S*. *cerevisiae*, bearing the empty vector plasmid or the Drk1 gene were cultured overnight and back-passed as above. After 3 hours of induction with 2% galactose, either DMSO was added to a final concentration of 0.1% or fludioxonil in DMSO was added to 10 μg/ml and 0.1% respectively (DMSO vehicle was minimized in this assay due to its effect on background MG stress). After 15 min, 1 hour and 3 hours of fludioxonil exposure, yeast were collected, washed once in H_2_O and suspended in 0.5 M perchloric acid containing 10 mM 1,2-diaminobenzene (DAB). Yeast were lysed by vortexing with glass beads as described above. Lysed yeast and denatured proteins were pelleted and supernates were retained. Supernates were incubated at RT for 30 min to allow methylglyoxal (MG) to form the 2-methylquinoxaline (MQ) adduct with DAB^49^. Quinoxalines were extracted using 500 mg hypersep C18 columns activated with 2.5 mls of methanol. After sample addition, columns were washed twice with 2.5 mls of ammonium phosphate buffer, (20 mM, pH 2.3) and quinoxalines were eluted with 2.5 mls of methanol. Methanol was evaporated under vacuum in a centrivap concentrator (Labconco) and quinoxalines were re-suspended in mobile phase for HPLC (40% Ammonium formate, 25 mM, pH 3.4, 60% methanol. 20 μl of each sample was applied to a LiChroCART 250-2 HPLC column containing Purospher STAR RP-18 attached to a System Gold 126 (Beckman Coulter) HPLC apparatus. Methylquinoxaline adduct was detected with a System Gold 168 detector at 320 nM and peaks were analyzed in 32 Karat software (Beckman Coulter, ver.5).

### DHAP determination *in vivo* and MG *in vitro*

DHAP *in vivo* was determined using a DHAP assay kit (Abcam), as per the accompanying protocols. Production of MG by TPI reaction *in vitro* used this kit with the addition of 1 mM DAB. Methylquinoxaline adduct was then detected by OD^320^ on a Genesys 20 spectrophotometer (Thermo Scientific).

### Fludioxonil Docking in Triosephosphate Isomerase

Small molecules were built in Sybyl (Certara Corp.) and saved in mol2 format. The two TPI protein receptors from *Trypanosoma cruzi* (PDB code 1tcd) and *S*. *cerevisiae* (1ypi) were prepared in Sybyl using the protein preparation tools to add hydrogens, properly type atoms, and remove any clashes. AutoDock Tools^86^ were used to prepare the ligand and receptor pdbqt files and to select the docking box size for Autodock4. The docking box size was large enough to include the dimer interface described by Kurkcuoglu *et al*.^50^. Autodock4.2.6 was run with Lamarkian Genetic Algorithm, with 25 M energy evaluations for 27,000 generations. This method includes a Solis & Wets local search of the ligand in the receptor after docking. Autodock 4.2.6 calculates a binding energy by summation of the molecular energy components (vdw + Hbonding + desolvation + electrostatics + ligand torsional free energy) minus the unbound system energy. The 30 best-docked ligands were examined. Optimal docking results were displayed in Pymol (PyMOL Mol. Graphics System, Ver. 1.8.2, Schrodinger, LLC).

## Supplementary information

Supplemental information

## References

[1] EPA-HQ-OPP-2010-1067. (EPA docket, Regulations.gov, 2011).

[2] Kilani J, Fillinger S (2016). Phenylpyrroles: 30 Years, Two Molecules and (Nearly) No Resistance. Front Microbiol.

[3] Arima K, Imanaka H, Kousaka M, Fukuta A, Tamura G (1964). Pyrrolnitrin, a New Antibiotic Substance, Produced by Pseudomonas. Agricultural and Biological Chemistry.

[4] Gehmann, K., Nyfeler, R., Leadbeater, A. J., Nevill, D. J. & Sozzi, D. In *Brighton Crop Protection Conference*, *Pests and Diseases - 1990*. *Vol*. *2*. (1990).

[5] Defosse TA (2015). Hybrid histidine kinases in pathogenic fungi. Molecular microbiology.

[6] Viaud M (2006). A class III histidine kinase acts as a novel virulence factor in Botrytis cinerea. Mol Plant Microbe Interact.

[7] Bahn YS (2007). Sensing the environment: lessons from fungi. Nat Rev Microbiol.

[8] Catlett NL, Yoder OC, Turgeon BG (2003). Whole-genome analysis of two-component signal transduction genes in fungal pathogens. Eukaryot Cell.

[9] Ota IM, Varshavsky A (1993). A yeast protein similar to bacterial two-component regulators. Science.

[10] Posas F (1996). Yeast HOG1 MAP kinase cascade is regulated by a multistep phosphorelay mechanism in the SLN1-YPD1-SSK1 “two-component” osmosensor. Cell.

[11] Saito H, Tatebayashi K (2004). Regulation of the osmoregulatory HOG MAPK cascade in yeast. J Biochem.

[12] Horie T, Tatebayashi K, Yamada R, Saito H (2008). Phosphorylated Ssk1 prevents unphosphorylated Ssk1 from activating the Ssk2 mitogen-activated protein kinase kinase kinase in the yeast high-osmolarity glycerol osmoregulatory pathway. Mol Cell Biol.

[13] Buschart A (2012). A novel functional assay for fungal histidine kinases group III reveals the role of HAMP domains for fungicide sensitivity. J Biotechnol.

[14] Kojima K (2004). Fungicide activity through activation of a fungal signalling pathway. Molecular microbiology.

[15] Furukawa K (2007). Novel reporter gene expression systems for monitoring activation of the Aspergillus nidulans HOG pathway. Bioscience, biotechnology, and biochemistry.

[16] Motoyama T (2005). A two-component histidine kinase of the rice blast fungus is involved in osmotic stress response and fungicide action. Fungal genetics and biology: FG & B.

[17] Lawry, S. M. *et al*. Fludioxonil Induces Drk1, a Fungal Group III Hybrid Histidine Kinase, To Dephosphorylate Its Downstream Target, Ypd1. *Antimicrobial agents and chemotherapy* **61**, 10.1128/AAC.01414-16 (2017). 10.1128/AAC.01414-16 PMC5278731 27872062

[18] Motoyama T (2005). An Os-1 family histidine kinase from a filamentous fungus confers fungicide-sensitivity to yeast. Curr Genet.

[19] Furukawa, K., Randhawa, A., Kaur, H., Mondal, A. K. & Hohmann, S. Fungal fludioxonil sensitivity is diminished by a constitutively active form of the group III histidine kinase. *FEBS letters*, 10.1016/j.febslet.2012.05.057 (2012). 10.1016/j.febslet.2012.05.057 22687241

[20] Kaur, H. *et al*. Differential Role of HAMP-like Linkers in Regulating the Functionality of the Group III Histidine Kinase DhNik1p. *The Journal of biological chemistry*, 10.1074/jbc.M114.554303 (2014). 10.1074/jbc.M114.554303 PMC5396353 24895133

[21] Meena N, Kaur H, Mondal AK (2010). Interactions among HAMP Domain Repeats Act as an Osmosensing Molecular Switch in Group III Hybrid Histidine Kinases from Fungi. Journal of Biological Chemistry.

[22] Wong J, Chen Y, Gan YH (2015). Host Cytosolic Glutathione Sensing by a Membrane Histidine Kinase Activates the Type VI Secretion System in an Intracellular Bacterium. Cell Host Microbe.

[23] Hancock J (2006). Doing the unexpected: proteins involved in hydrogen peroxide perception. Journal of experimental botany.

[24] Pompliano DL, Peyman A, Knowles JR (1990). Stabilization of a reaction intermediate as a catalytic device: definition of the functional role of the flexible loop in triosephosphate isomerase. Biochemistry.

[25] Maeda T, Wurgler-Murphy SM, Saito H (1994). A two-component system that regulates an osmosensing MAP kinase cascade in yeast. Nature.

[26] Tebbets B (2012). Identification and characterization of antifungal compounds using a Saccharomyces cerevisiae reporter bioassay. PloS one.

[27] Wong DT, Horng JS, Gordee RS (1971). Respiratory chain of a pathogenic fungus, Microsporum gypseum: effect of the antifungal agent pyrrolnitrin. Journal of bacteriology.

[28] Yano T (2010). A novel fluorescent sensor protein for visualization of redox states in the cytoplasm and in peroxisomes. Mol Cell Biol.

[29] Oku M, Hoseki J, Ichiki Y, Sakai Y (2013). A fluorescence resonance energy transfer (FRET)-based redox sensor reveals physiological role of thioredoxin in the yeast Saccharomyces cerevisiae. FEBS letters.

[30] Shah I (2016). Using ToxCast™ Data to Reconstruct Dynamic Cell State Trajectories and Estimate Toxicological Points of Departure. Environ Health Perspect.

[31] Kavlock R (2012). Update on EPA’s ToxCast program: providing high throughput decision support tools for chemical risk management. Chem Res Toxicol.

[32] Lambowitz AM, Slayman CW (1972). Effect of pyrrolnitrin on electron transport and oxidative phosphorylation in mitochondria isolated from Neurospora crassa. Journal of bacteriology.

[33] Lee HC, Yin PH, Lu CY, Chi CW, Wei YH (2000). Increase of mitochondria and mitochondrial DNA in response to oxidative stress in human cells. The Biochemical journal.

[34] Wredenberg A (2002). Increased mitochondrial mass in mitochondrial myopathy mice. Proceedings of the National Academy of Sciences of the United States of America.

[35] M’Bemba-Meka P, Lemieux N, Chakrabarti SK (2006). Role of oxidative stress, mitochondrial membrane potential, and calcium homeostasis in nickel subsulfide-induced human lymphocyte death *in vitro*. Sci Total Environ.

[36] Lazarova N, Krumova E, Stefanova T, Georgieva N, Angelova M (2014). The oxidative stress response of the filamentous yeast Trichosporon cutaneum R57 to copper, cadmium and chromium exposure. Biotechnol Biotechnol Equip.

[37] Hosiner D (2014). Impact of acute metal stress in Saccharomyces cerevisiae. PloS one.

[38] Nelson KJ (2010). Use of dimedone-based chemical probes for sulfenic acid detection methods to visualize and identify labeled proteins. Methods Enzymol.

[39] Golla U, Bandi G, Tomar RS (2015). Molecular cytotoxicity mechanisms of allyl alcohol (acrolein) in budding yeast. Chem Res Toxicol.

[40] Wonisch W, Schaur RJ, Bilinski T, Esterbauer H (1995). Assessment of growth inhibition by aldehydic lipid peroxidation products and related aldehydes by Saccharomyces cerevisiae. Cell Biochem Funct.

[41] Turton HE, Dawes IW, Grant CM (1997). Saccharomyces cerevisiae exhibits a yAP-1-mediated adaptive response to malondialdehyde. Journal of bacteriology.

[42] Cai J, Bhatnagar A, Pierce WM (2009). Protein modification by acrolein: formation and stability of cysteine adducts. Chem Res Toxicol.

[43] Maeta K, Izawa S, Inoue Y (2005). Methylglyoxal, a metabolite derived from glycolysis, functions as a signal initiator of the high osmolarity glycerol-mitogen-activated protein kinase cascade and calcineurin/Crz1-mediated pathway in Saccharomyces cerevisiae. J Biol Chem.

[44] Inoue Y, Maeta K, Nomura W (2011). Glyoxalase system in yeasts: structure, function, and physiology. Semin Cell Dev Biol.

[45] Compagno C (2001). Alterations of the glucose metabolism in a triose phosphate isomerase-negative Saccharomyces cerevisiae mutant. Yeast.

[46] Lo TW, Westwood ME, McLellan AC, Selwood T, Thornalley PJ (1994). Binding and modification of proteins by methylglyoxal under physiological conditions. A kinetic and mechanistic study with N alpha-acetylarginine, N alpha-acetylcysteine, and N alpha-acetyllysine, and bovine serum albumin. The Journal of biological chemistry.

[47] Maeta K, Izawa S, Okazaki S, Kuge S, Inoue Y (2004). Activity of the Yap1 transcription factor in Saccharomyces cerevisiae is modulated by methylglyoxal, a metabolite derived from glycolysis. Mol Cell Biol.

[48] Antelmann H, Helmann JD (2011). Thiol-based redox switches and gene regulation. Antioxid Redox Signal.

[49] Cordeiro C, Ponces Freire A (1996). Methylglyoxal assay in cells as 2-methylquinoxaline using 1,2-diaminobenzene as derivatizing reagent. Analytical biochemistry.

[50] Kurkcuoglu Z, Findik D, Akten ED, Doruker P (2015). How an Inhibitor Bound to Subunit Interface Alters Triosephosphate Isomerase Dynamics. Biophys J.

[51] Espinoza-Fonseca LM, Trujillo-Ferrara JG (2005). Structural considerations for the rational design of selective anti-trypanosomal agents: the role of the aromatic clusters at the interface of triosephosphate isomerase dimer. Biochemical and biophysical research communications.

[52] Jespers ABK, Dewaard MA (1995). Effect of Fenpiclonil on Phosphorylation of Glucose in Fusarium-Sulphureum. Pestic Sci.

[53] Ochiai N (2002). Effects of iprodione and fludioxonil on glycerol synthesis and hyphal development in Candida albicans. Biosci Biotechnol Biochem.

[54] Ray S, Biswas S, Ray M (1997). Similar nature of inhibition of mitochondrial respiration of heart tissue and malignant cells by methylglyoxal. A vital clue to understand the biochemical basis of malignancy. Mol Cell Biochem.

[55] Biswas S, Ray M, Misra S, Dutta DP, Ray S (1997). Selective inhibition of mitochondrial respiration and glycolysis in human leukaemic leucocytes by methylglyoxal. The Biochemical journal.

[56] Roy A (2016). The glucose metabolite methylglyoxal inhibits expression of the glucose transporter genes by inactivating the cell surface glucose sensors Rgt2 and Snf3 in yeast. Mol Biol Cell.

[57] Yoshida A, Wei D, Nomura W, Izawa S, Inoue Y (2012). Reduction of glucose uptake through inhibition of hexose transporters and enhancement of their endocytosis by methylglyoxal in Saccharomyces cerevisiae. The Journal of biological chemistry.

[58] Brandes N, Schmitt S, Jakob U (2009). Thiol-based redox switches in eukaryotic proteins. Antioxid Redox Signal.

[59] Liu W, Leroux P, Fillinger S (2008). The HOG1-like MAP kinase Sak1 of Botrytis cinerea is negatively regulated by the upstream histidine kinase Bos1 and is not involved in dicarboximide- and phenylpyrrole-resistance. Fungal genetics and biology: FG & B.

[60] Kim JH (2007). Enhancement of fludioxonil fungicidal activity by disrupting cellular glutathione homeostasis with 2,5-dihydroxybenzoic acid. FEMS microbiology letters.

[61] Kanetis L, Forster H, Jones CA, Borkovich KA, Adaskaveg JE (2008). Characterization of genetic and biochemical mechanisms of fludioxonil and pyrimethanil resistance in field isolates of Penicillium digitatum. Phytopathology.

[62] Cui W, Beever RE, Parkes SL, Templeton MD (2004). Evolution of an Osmosensing Histidine Kinase in Field Strains of Botryotinia fuckeliana (Botrytis cinerea) in Response to Dicarboximide Fungicide Usage. Phytopathology.

[63] Ren W, Shao W, Han X, Zhou M, Chen C (2016). Molecular and Biochemical Characterization of Laboratory and Field Mutants of Botrytis cinerea Resistant to Fludioxonil. Plant Disease.

[64] Kim HJ, Ha S, Lee HY, Lee KJ (2015). ROSics: chemistry and proteomics of cysteine modifications in redox biology. Mass Spectrom Rev.

[65] Araki K (2016). Redox Sensitivities of Global Cellular Cysteine Residues under Reductive and Oxidative Stress. J Proteome Res.

[66] Tripathi RK, Gottlieb D (1969). Mechanism of action of the antifungal antibiotic pyrrolnitrin. Journal of bacteriology.

[67] Wong DT, Airall JM (1970). The mode of action of antifungal agents: effect of pyrrolnitrin on mitochondrial electron transport. The Journal of antibiotics.

[68] Grek CL, Zhang J, Manevich Y, Townsend DM, Tew KD (2013). Causes and consequences of cysteine S-glutathionylation. The Journal of biological chemistry.

[69] Marsh L, Shah K (2014). A novel inhibitor of Mammalian triosephosphate isomerase found by an in silico approach. Int J Med Chem.

[70] Aguilera E (2016). Potent and Selective Inhibitors of Trypanosoma cruzi Triosephosphate Isomerase with Concomitant Inhibition of Cruzipain: Inhibition of Parasite Growth through Multitarget Activity. ChemMedChem.

[71] Alvarez G (2010). Massive screening yields novel and selective Trypanosoma cruzi triosephosphate isomerase dimer-interface-irreversible inhibitors with anti-trypanosomal activity. Eur J Med Chem.

[72] Bonnet R, Pavlovic S, Lehmann J, Rommelspacher H (2004). The strong inhibition of triosephosphate isomerase by the natural beta-carbolines may explain their neurotoxic actions. Neuroscience.

[73] Martins AM, Cordeiro CA, Ponces Freire AM (2001). *In situ* analysis of methylglyoxal metabolism in Saccharomyces cerevisiae. FEBS letters.

[74] Li N (2003). Mitochondrial complex I inhibitor rotenone induces apoptosis through enhancing mitochondrial reactive oxygen species production. J Biol Chem.

[75] Coleman MD (2012). A preliminary investigation into the impact of a pesticide combination on human neuronal and glial cell lines *in vitro*. PloS one.

[76] Teng Y (2013). Endocrine disruptors fludioxonil and fenhexamid stimulate miR-21 expression in breast cancer cells. Toxicological sciences: an official journal of the Society of Toxicology.

[77] Brandhorst, T. T. & Klein, B. S. Uncertainty surrounding the mechanism and safety of the post-harvest fungicide fludioxonil. *Food Chem Toxicol*, 10.1016/j.fct.2018.11.037 (2018). 10.1016/j.fct.2018.11.037 PMC6322420 30458269

[78] Green SR, Moehle CM (2001). Media and culture of yeast. Curr Protoc Cell Biol.

[79] Dower WJ, Miller JF, Ragsdale CW (1988). High efficiency transformation of E. coli by high voltage electroporation. Nucleic acids research.

[80] Gietz RD, Schiestl RH (2007). Quick and easy yeast transformation using the LiAc/SS carrier DNA/PEG method. Nat Protoc.

[81] Basu S, Wang X, Gladwin MT, Kim-Shapiro DB (2008). Chemiluminescent detection of S-nitrosated proteins: comparison of tri-iodide, copper/CO/cysteine, and modified copper/cysteine methods. Methods Enzymol.

[82] Kallberg M (2012). Template-based protein structure modeling using the RaptorX web server. Nat Protoc.

[83] Marchler-Bauer A (2015). CDD: NCBI’s conserved domain database. Nucleic acids research.

[84] Schindelin J (2012). Fiji: an open-source platform for biological-image analysis. Nat Methods.

[85] Esnouf MP, Harris RP, McVittie JD (1982). Triosephosphate isomerase from chicken and rabbit muscle. Methods Enzymol.

[86] Morris GM (2009). AutoDock4 and AutoDockTools4: Automated docking with selective receptor flexibility. J Comput Chem.

[87] Bourret RB (2010). Receiver domain structure and function in response regulator proteins. Current opinion in microbiology.

