# Supplementary material for: Phenylpyrrole fungicides act on triosephosphate isomerase to induce methylglyoxal stress and alter hybrid histidine kinase activity
Source: Sci Rep. 2019 Mar 25;9:5047. doi: 10.1038/s41598-019-41564-9 (PMC6433957; doi:10.1038/s41598-019-41564-9)
Supplement: Supplementary file 1 — Supplemental information [file 41598_2019_41564_MOESM1_ESM.pdf]

SUPPLEMENTAL INFORMATION

Paper Title: Phenylpyrrole fungicides act on triosephosphate isomerase to induce methylglyoxal stress and alter hybrid histidine kinase activity

Authors: T Tristan Brandhorst, Iain Kean, Stephanie Lawry, Darin Wiesner and Bruce Klein

Contents:

Supplementary Figure S2 Results, Discussion and References

Six Supplemental figures and legends

Three Supplemental Tables

Supplementary Figure S2 data and Discussion

Some fludioxonil-like leads we identified bore a resemblance to known nitrosoglutathione reductase (GSNOR) inhibitors. Nitrosoglutathione (GSNO) comprises the intracellular pool of nitric oxide, and can serve as a signaling molecule by nitrosating or glutathionylating sensitive sentinel cysteine thiols within sensor kinases, inducing perturbations in their structure and function[1,2]. GSNOR breaks down GSNO[1], so, if fludioxonil were to inhibit GSNOR, this could translate into an accumulation of thiol-modifying GSNO in the cell.

To investigate a model in which fludioxonil toxicity hinges upon GSNOR inhibition, we looked first at whether fludioxonil could inhibit GSNOR activity. We used mouse liver lysate (a common source of mammalian GSNOR[3]), and recombinant purified human GSNOR (rGSNOR). Murine and human GSNOR have 66% and 63% identity with yeast GSNOR (NCBI Protein Blast[4]), and were more available than yeast GSNOR. We observed up to 43% and 56% inhibition of GSNOR activity in murine liver lysate and rGSNOR, respectively, but it took a high concentration, 40μg/ml, of fludioxonil to achieve this level of inhibition **(Supplementary Fig. S2)**. In contrast, N6022, a known GSNOR inhibitor[5], reduced GSNOR activity in murine liver lysate nearly 100% at 10μg/ml.

As a corollary of GSNOR inhibition, we tested whether fludioxonil increases nitrosation in the cell. We measured the levels of S-nitrosothiols (SNOs) in the cell with a nitric oxide analyzer. This instrument identifies both protein S-nitrosation and SNO pools such as GSNO. Because levels of nitrosation are relatively low in yeast, as compared to mammalian cells, we added exogenous GSNO to increase baseline nitrosation to a detectable level. We analyzed nitric oxide levels in wild-type *S. cerevisiae* after exposure to either fludioxonil or the known GSNOR inhibitor N6022[5]. With N6022 treatment, SNO increased by nearly one log (15,004 vs. 119,840) over GSNO treatment alone **(Supplementary Fig. S2)**. Fludioxonil treatment, on the other hand, did not increase the levels of SNO. In fact, fludioxonil treatment lead to a roughly

50% decrement in SNO levels (15,004 vs. 7,363), although this change was not statistically significant given the low concentration of nitrosothiols involved.

To test definitively whether inhibition of GSNOR activity is required for the action of fludioxonil, we determined whether yeast deficient in GSNOR are resistant to fludioxonil. Using wild type or GSNOR knockout (KO) yeast, each containing either Drk1 or empty vector as a control, we assayed the effect of GSNOR deletion on sensitivity to fludioxonil **(Supplementary Fig. S2)**. Upon growth in glucose (no Drk1 expression), none of the strains were sensitive to fludioxonil. When grown in galactose (Drk1 expression), both wild-type and GSNOR KO yeast expressing Drk1 were similarly sensitive to the drug, indicating that the capacity to inhibit GSNOR does not impact fludioxonil sensitivity. Thus, while high concentrations of fludioxonil modestly affected GSNOR activity *in vitro*, GSNOR is dispensable in the mode of drug action. These data, taken together with the absence of elevated nitrosothiols in fludioxonil treated yeast, do not support a model in which Drk1 is converted from a kinase to a phosphatase by the reaction of cysteine thiols with nitrosothiols.

Glutathionylation of Drk1 does not take place in response to Fludioxonil exposure

While GSNOR inhibition and Drk1 nitrosation are not required for the mechanism of action of fludioxonil, cysteine residues are also responsive to glutathionylation[1,2]. Glutathionylation of cysteine thiols is sufficiently stable that this modification may be quantified in affected proteins. For mass spectroscopy (MS) analysis, thiols were blocked and glutathione removed from thiols enzymatically using glutaredoxin prior to labelling with iotoacetic acid (IAA). Whereas glutathionylated cysteine residues were detectable in Drk1 in untreated yeast (certain cysteines exhibited a background level of glutathionylation), there was no increase in glutathionylation of any thiols of Drk1 in response to fludioxonil treatment (data not shown). Thus, the mode of fludioxonil toxicity likely does not hinge on glutathionylation of Drk1 cysteines.

1 Broniowska, K. A., Diers, A. R. & Hogg, N. S-nitrosoglutathione. *Biochimica et biophysica acta* **1830**, 3173-3181, doi:10.1016/j.bbagen.2013.02.004 (2013).

2 Konorev, E. A., Kalyanaraman, B. & Hogg, N. Modification of creatine kinase by S-nitrosothiols: S-nitrosation vs. S-thiolation. *Free radical biology & medicine* **28**, 1671-1678 (2000).

3 Tsuboi, S. *et al.* Purification and characterization of formaldehyde dehydrogenase from rat liver cytosol. *J Biochem* **111**, 465-471 (1992).

4 Altschul, S. F., Gish, W., Miller, W., Myers, E. W. & Lipman, D. J. Basic local alignment search tool. *Journal of molecular biology* **215**, 403-410, doi:10.1016/S0022-2836(05)80360-2 (1990).

5 Sun, X. *et al.* Discovery of s-nitrosoglutathione reductase inhibitors: potential agents for the treatment of asthma and other inflammatory diseases. *ACS Med Chem Lett* **2**, 402-406, doi:10.1021/ml200045s (2011).

Supplementary Figure S1

Drk1-dependent Fungicides

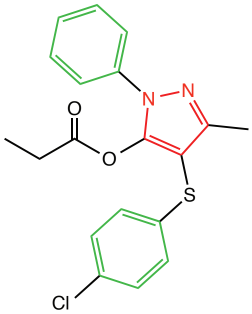

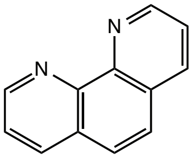

o-phenanthroline

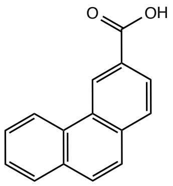

Known GSNOR Inhibitors

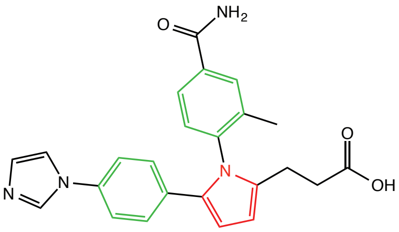

N6022

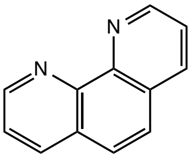

o-phenanthroline

Supplementary Figure S1. Compounds identified in a small molecule screen to detect Drk1-dependent fungicides share structural features with known GSNOR inhibitors. Many of these compounds (left) are heterocyclic pyrroles (red) with 2-3 substitutions, often aromatic rings (green), similar to the known GSNOR inhibitor N6022. Benzyl groups are attached, usually by a single carbon-carbon bond that allows the aromatic groups to adopt a co-planar orientation Our screen also showed that o-phenanthroline, a known GSNOR inhibitor, had fungicidal activity against Drk-1 expressing yeast.

Supplementary Figure S2

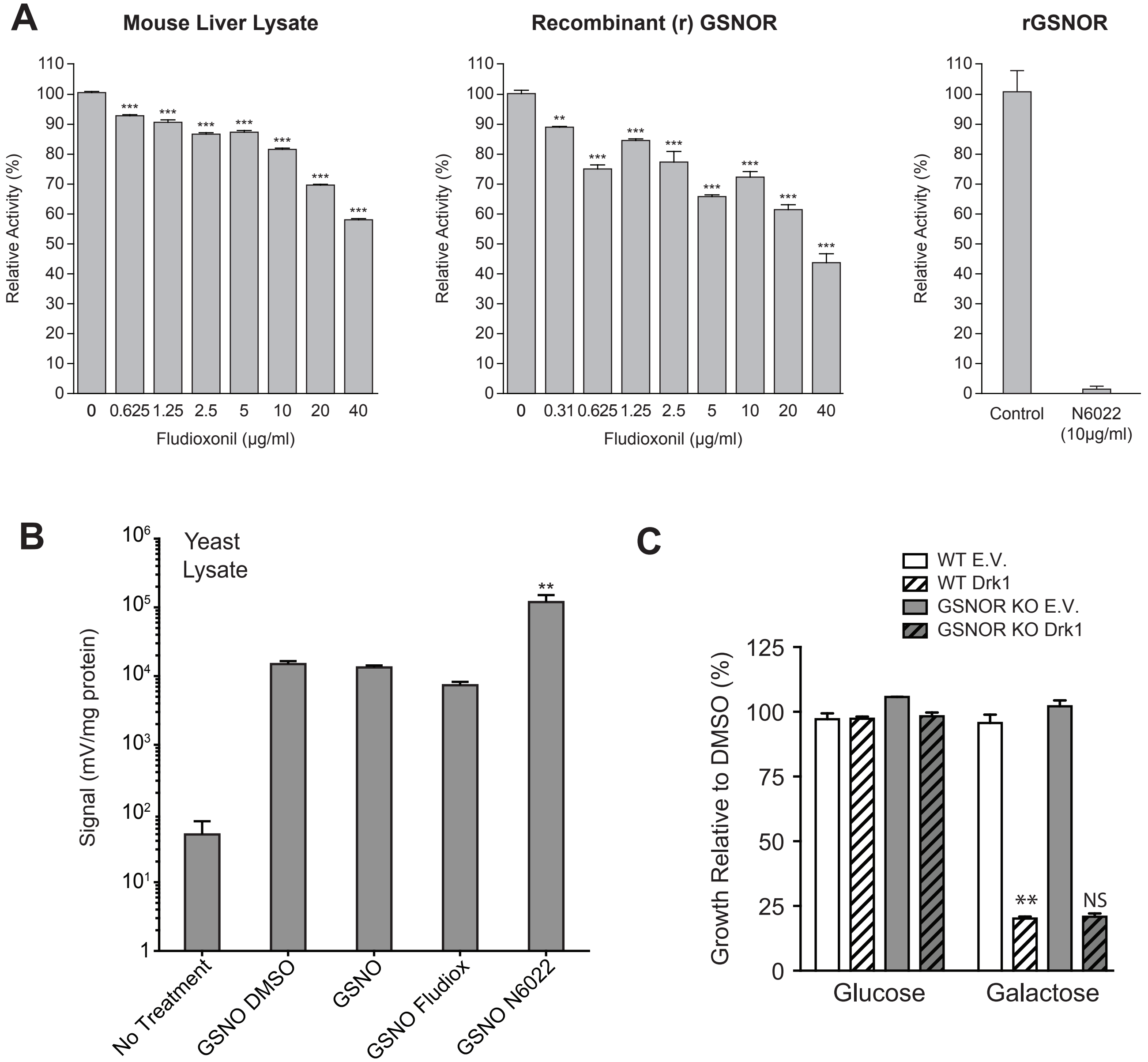

Supplementary Figure S2. Fludioxonil and nitrosative stress

(A) GSNOR activity of murine liver lysate or purified recombinant human GSNOR exposed to various concentrations of fudioxonil or N6022 inhibitor. Relative data is determined by dividing ΔA340 of the fludioxonil or inhibitor exposure by the ΔA340 of DMSO alone. Data are mean ± SEM of triplicates. **, $p<0.01$, ***, $p<0.001$, compared to 0µg/ml based on a one-way ANOVA with a Dunnets multiple comparison post-test.

(B) SNO levels (including small molecular weight SNOs like GSNO as well as protein nitrosation) in S. cerevisiae after 2 hours of growth alone (no treatment), with 5mM GSNO, or with 5mM GSNO and either 1% DMSO, 25µg/ml fludioxonil,or 25µg/ml N6022, a known GSNOR inhibitor. Data are the mean of two technical replicates for each of two biological replicates ± SEM. **: $p<0.01$ compared to all other samples based on one way ANOVA with a Tukey post-test.

(C) Growth of wild-type or GSNOR knockout of *S. cerevisiae* harboring either empty vector (EV) or Drk1 under the control of a galactose-inducible promoter after growth at 30°C in a 96-well plate. Growth is reported as the relative growth in 25µg/ml fludioxonil in 1% DMSO compared to growth in 1% DMSO alone. **, $p< 0.01$ vs. WT; Analysis by two-way ANOVA with Tukey post-test. NS, not significant (vs. GSNOR KO E.V. control).

Supplementary Figure S3

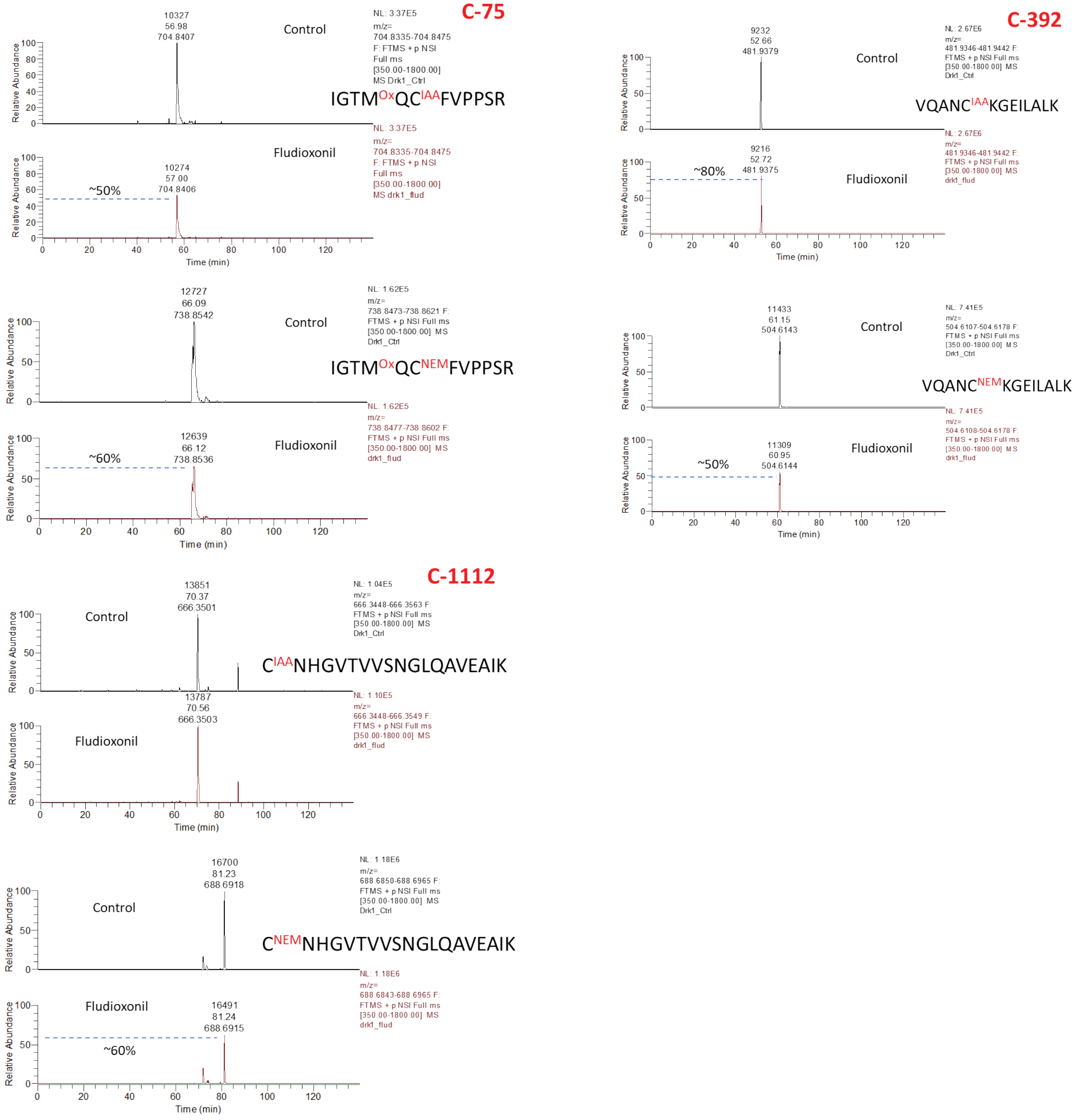

Supplementary Figure S3. MS analysis of Cysteine modification before and after fludioxonil exposure

The reduced cysteines of Drk1 were labeled with NEM, while DTT reduceable cysteines were subsequently labelled with IAA.The lack of an increase in the abundance of IAA labelled cysteines indicates that none of them become dimerized during a stress response to fludioxonil. In the case of cysteines C-75, C-392, and C-1112, the abundance of reduced cyteines was diminished following fludioxonil treatment. These cysteines were neither reduced nor were they reduceable, indicating that a more stable modification of the thiol has taken place as a consequence of fluidioxonil treatment.

Supplementary Figure S4

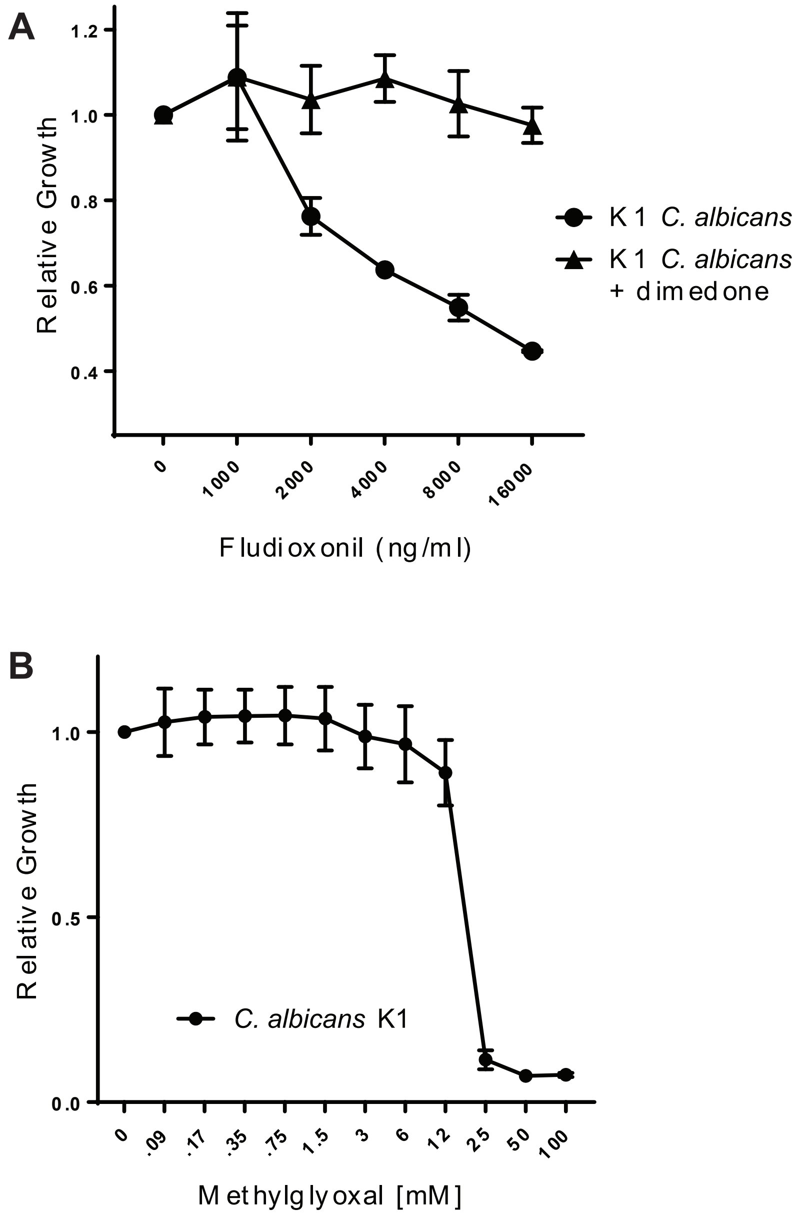

Supplementary Figure S4. The attenuation of fludioxonil toxicity, and sensitivity to MG, are also observed in *C. albicans* strain K1

(A) Growth assay measuring the effect of the aldehyde binding molecule dimedone on fludioxonil killing of *C. albicans* strain K1. Dimedone is added at 1mM concentration. Growth relative to DMSO controls is shown. (B) Growth inhibition effect of the aldehyde methylglyoxal upon the fludioxonil sensitive *C. albicans* strain K1

Supplemenatary Figure S5

A

Coverage of tryptic digest of Drk1 from *S. cerevisea*

```
   1 MTRGDETLLA VAGILQGLAK DVPDSASLPF NSYKSNNATN GDVAKINLPG
  51 ENSDGKAVLE RELEDLIRRI GTMQCFVPPS RRSTRAAISS GQDTNSHGSP
 101 KPLPGSDVNY EDDIHFLQNR VQLQAQEIQL QKDVISRVRE ELKEQEKNTE
 151 KALGKVKKED VGILERELRK HQQANEAFQK ALREIGGIIT QVANGDLSMK
 201 VQIHPLEMDP EITTFKRTIN TMMDQLQVFG SEVSRVAREV GTEGILGGQA
 251 QITGVHGIWK ELTENVNIMA KNLTDQVREI ATVTTAVAHG DLSQKIESRA
 301 KGEILELQQT INTMVDQLRT FATEVTRVAR DVGTEGVLGG QAQIEGVQGM
 351 WNELTVNVNA MAENLTTQVR DIAMVTTAVA KGDLTQKVQA NCKGEILALK
 401 TIINSMVDQL KQFAQEVTKI AKEVGTDGVL GGQATVHDVE GTWKDLTENV
 451 NGMAMNLTTQ VREIADVTTA VAKGDLTKKV TADVKGEILD LKNTINGMVD
 501 RLNTFAFEVS KVAREVGTDG TLGGQAKVDN VEGKWKDLTD NVNTMAQNLT
 551 SQVRGISEVT QAIAKGELAK KIEVHAQGEI LTLKVTINNM VDRLANFAHE
 601 LKRVARDVGV DGKMGGQANV EGIAGRWKEI TEDVNTMAEN LTSQVRAFGE
 651 ITDAATDGDF TKLITVNASG EMDELKRKIN KMVSNLRDSI QRNTAAREAA
 701 ELANRTKSEF LANMSHEIRT PMNGIIGMTQ LTLDTDDLKP YPREMLNVVH
 751 SLANSLLTII DDILDISKIE ANRMVIEKIP FSMRGTVFNA LKTLAVKANE
 801 KFLSLAYQVD SSVPDYVTGD PFRLRQIILN LVGNAIKFTE HGEVKLAISR
 851 SDREECKDNE YAFEFSVSDT GIGIEEDKLD LIFDTFQQAD GSTTRKFGGT
 901 GLGLSISKRL VNLMGGDVWV TSEYGLGSSF HFTCVVELAD QSISMISASL
 951 MPYKNHRVLF IDKGQTGGHA EEITEMLKQL DLEPIVVKDE SQVPPPEIQD
1001 PTGKDSGHAY DVIIVDSVDT ARNLRTYDEF KYIPIVLLCP VVSVSLKSAL
1051 DLGITSYMTT PCQPIDLGNG MLPALEGRST PITTDHTRSF DILLAEDNDV
1101 NQRVAVKILE KCNHGVTVVS NGLQAVEAIK KRRYDVILMD VQMPVMGGFE
1151 ATGKIREYEK KNGLSRTPII ALTAHAMLGD REKCIQAQMD EYLAKPLKQN
1201 QMIQTILKCA TLGGSLLEKS KEPRMSSSGE PPHHVPNSNG MKSLDTKNQR
1251 PGMDSQATSA SGGPNPNQKS DVVSTRQTRV ASSWTK
```

B

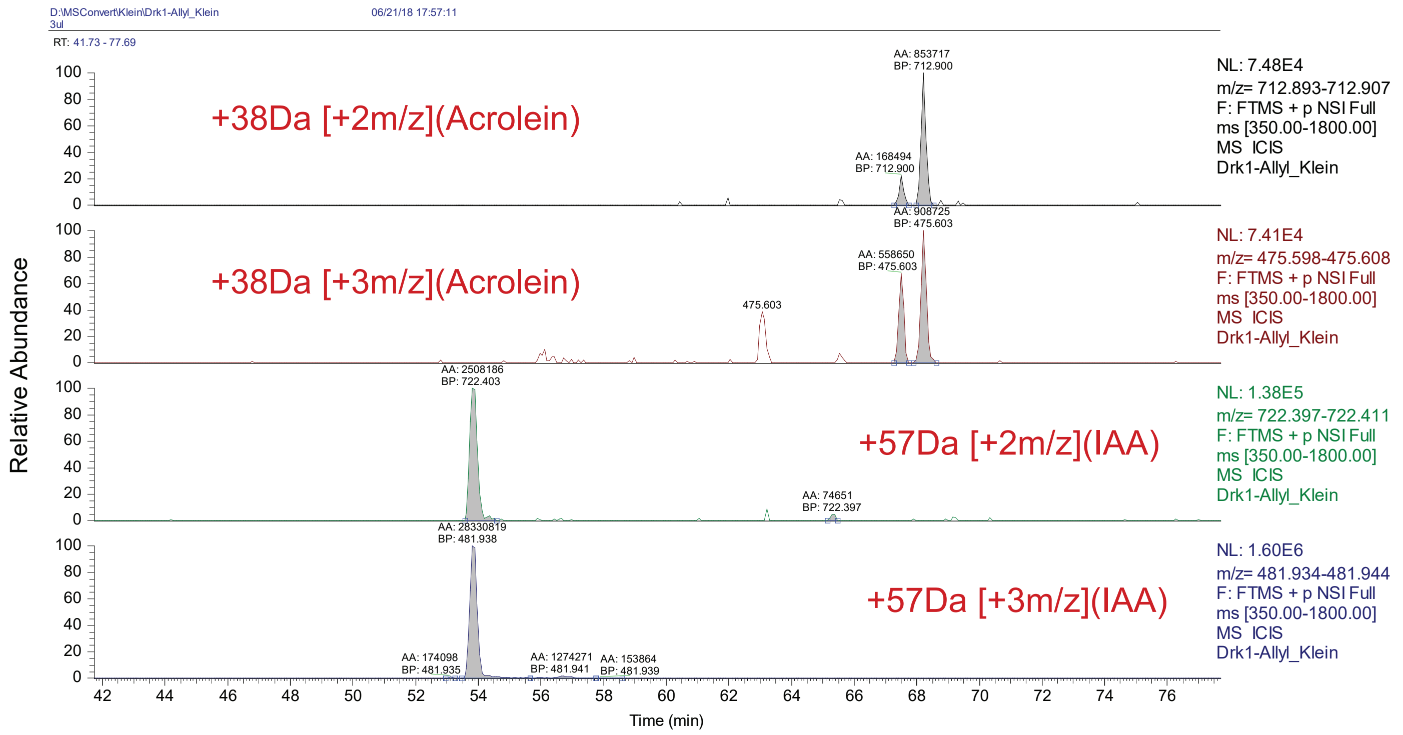

Supplementary Figure S5. Tryptic peptides of Drk1 HHK from *S. cerevisiae*

A) Sequence of peptides was analyzed by nanoLC-MS/MS. Fragments containing cysteines at positions 75, 392,1039,1062, 1112, 1184, and 1209 are represented. Red denotes residues discerned on peptide fragments.Two cysteine containing peptides were not detectable at positions 856 and 934.

B) Relative abundance of modified vs. IAA labeled (unmodified) were calculated by aggregating the volumes of representative peaks comprising 95% of total signal and receiving high (high confidence) ion scores. C392 fragment shown as an example.

Supplementary Figure S6

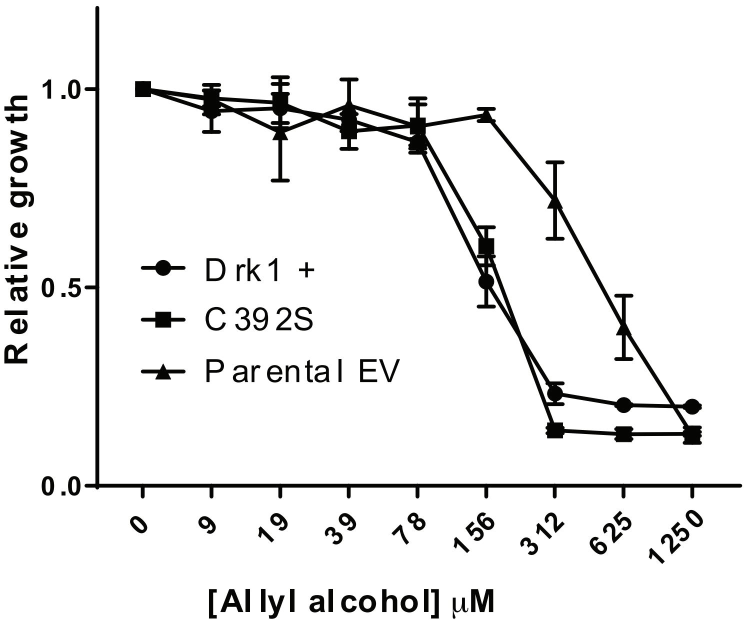

Supplementary Figure S6. Growth assay measuring the effect of allyl alcohol on C392S Drk1-expressing S. cerevisiae BY4741 compared to empty vector(EV)-transformed parental strain (negative control) and WT Drk1(positive control). Parental empty vector control is insensitive to fludioxonil, but succumbs to [allyl alcohol] > 156 mM. Despite a differential sensitivity to fludioxonil compared to WT Drk1, C392S Drk1 does not evidence a reduced sensitivity to allyl alcohol treatment. Growth relative to DMSO treatment is shown.

Table S1. Strains used in this study*

	Name	Background	Description/Plasmid(s)	Reference or Source
S. cerevisiae	WT BY4741	BY4741	*MAT***a** *his3Δ1 leu2Δ0 met15Δ0 ura3Δ0*	[86,87]
	Empty Vector	BY4741	WT transformed with pYES-DEST52 empty vector	[17]
	Drk1	BY4741	WT transformed with pYES-DEST52 Drk1 plasmid	[17]
	GSNOR KO	BY4741	Sfa1 Knockout	GE Dharmacon (#YSC6273-201935646)
	GSNOR KO EV	BY4741	WT transformed with pYES-DEST52 empty vector	This work
	GSNOR KO Drk1	BY4741	WT transformed with pYES-DEST52 Drk1 plasmid	This work
	Drk1 C75S	BY4741	WT transformed with pYES-DEST52 Drk1 C75S plasmid	This work
	Drk1 C392S	BY4741	WT transformed with pYES-DEST52 Drk1 C392S plasmid	This work
	Drk1 C856S	BY4741	WT transformed with pYES-DEST52 Drk1 C856S plasmid	This work
	Drk1 C934S	BY4741	WT transformed with pYES-DEST52 Drk1 C934S plasmid	This work
	Drk1 C1039S	BY4741	WT transformed with pYES-DEST52 Drk1 C1039S plasmid	This work
	Drk1 C1062S	BY4741	WT transformed with pYES-DEST52 Drk1 C1062S plasmid	This work
	Drk1 C1112S	BY4741	WT transformed with pYES-DEST52 Drk1 C1112S plasmid	This work
	Drk1 C1184S	BY4741	WT transformed with pYES-DEST52 Drk1 C1184S plasmid	This work
	Drk1 C1209S	BY4741	WT transformed with pYES-DEST52 Drk1 C1209S plasmid	This work
	Drk1 C392S/C856S	BY4741	WT transformed with pYES-DEST52 Drk1 C392S/C856S plasmid	This work
	YRF-1A	BY4741	WT transformed with pYRF-1A	[28]
	TM229		MATa ura3 leu2 his3 sln1-ts4	[25]
	TM229 Drk1	TM229	Sln1-ts strain transformed with pYES-DEST52 Drk1 plasmid	This work
E. coli	GST-Ypd1	DH10β	DH10β transformed with pGEX-KG-Ypd1	[17]
	GST-Sln1K	DH5α	DH5α transformed with pGEX-KG-Sln1K	[88]
	GST-Sln1R	DH5α	DH5α transformed with pGEX-KG-Sln1R	[88]
	GST-Drk1	BL21(DE3)	BL21(DE3) transformed with pGEX-KG-Drk1	[17]
Candida albicans	Strain K1	--	--	ATCC

*WT, wild-type

Table S2. Vectors used in this study

Name	Parent/Description	Reference or Source
pYES-DEST52 Empty Vector		Invitrogen Life Technologies
pYES-DEST52 Drk1	pYES-DEST52 with Drk1 insert	[17]
pYES-DEST52 Drk1 C75S	pYES-DEST52 Drk1 with Drk1 C75 mutated to serine	This work
pYES-DEST52 Drk1 C392S	pYES-DEST52 Drk1 with Drk1 C392 mutated to serine	This work
pYES-DEST52 Drk1 C856S	pYES-DEST52 Drk1 with Drk1 C856 mutated to serine	This work
pYES-DEST52 Drk1 C934S	pYES-DEST52 Drk1 with Drk1 C934 mutated to serine	This work
pYES-DEST52 Drk1 C1039S	pYES-DEST52 Drk1 with Drk1 C1039 mutated to serine	This work
pYES-DEST52 Drk1 C1062S	pYES-DEST52 Drk1 with Drk1 C1062 mutated to serine	This work
pYES-DEST52 Drk1 C1112S	pYES-DEST52 Drk1 with Drk1 C1112 mutated to serine	This work
pYES-DEST52 Drk1 C1184S	pYES-DEST52 Drk1 with Drk1 C1184 mutated to serine	This work
pYES-DEST52 Drk1 C1209S	pYES-DEST52 Drk1 with Drk1 C1209 mutated to serine	This work
pYES-DEST52 Drk1 C392S/C856S	pYES-DEST52 Drk1 with Drk1 C392 and C856 mutated to serine	This work
pYES-DEST52 Drk1 D1140N	pYES-DEST52 Drk1 with Drk1 D1140 mutated to asparagine	[17]
pGEX-KG-Ypd1 (pOU1)	pGEX-KG [89] with Ypd1 inserted with XbaI (5’) and XhoI (3’)	*
pGEX-KG-Sln1K (pHL581)	pGEX-KG with Sln1K (aa 537 to 947) insert	[88]†
pGEX-KG-Sln1R (pADA689)	pGEX-KG with Sln1R (aa 1068 to 1220) insert	[88]†
pYRF-1A	RedoxFluor construct plasmid	‡

*Plasmid was a generous gift from Ann West

† Plasmid was a generous gift from Jan Fassler

‡ Plasmid was a generous gift from Yoshi Sakai

Table S3. Primers used in this study

Name	Sequence (5' → 3')	Purpose/Target
SML166	GGATCGGTACTATGCAAAGTTTTGTGCCCCCATCC	Mutate C75 of Drk1 to serine
SML167	CAGAAAGTCCAGGCTAACAGCAAGGGTGAAATTCTTG	Mutate C392 of Drk1 to serine
SML168	CAGATCGGATCGAGAGGAAAGTAAAGATAATGAATACGC	Mutate C856 of Drk1 to serine
SML169	TAGCTTCCACTTCACGAGCGTTGTAGAACTGGC	Mutate C934 of Drk1 to serine
SML170	AGTTTAAATACATACCTATCGTTCTATTAAGCCCTGTCGTTTCTG	Mutate C1039 of Drk1 to serine
SML171	CGTACATGACAACTCCTAGTCAGCCTATCGATCTA	Mutate C1062 of Drk1 to serine
SML172	GCCGTCAAGATACTGGAGAAAAGCAACCATGGC	Mutate C1112 of Drk1 to serine
SML173	GGTGACCGGGAGAAAAGTATCCAAGCCCAGA	Mutate C1184 of Drk1 to serine
SML174	TCAAACCATCTTGAAGAGCGCAACTCTCGGTGG	Mutate C1209 of Drk1 to serine
IRLK099	CATTTTGATGGATGTCCAAATGC	Re-mutate the mutated N1140 in Drk1 D1140N back to aspartate
IRLK100	ACGTCGTATCGACGCTTC	Re-mutate the mutated N1140 in Drk1 D1140N back to aspartate
